# Biomimicry as a decision-making methodology in condition monitoring

**DOI:** 10.3389/frai.2025.1485489

**Published:** 2025-05-30

**Authors:** Hariom Dhungana

**Affiliations:** Department of Mechanical Engineering and Maritime Studies, Western Norway University of Applied Sciences, Bergen, Norway

**Keywords:** biomimicry, cognition, condition monitoring, predictive maintenance, safety, security

## Abstract

In maintenance engineering, effective decision-making is critical to ensuring system reliability and operational efficiency. Modern industrial systems are monitored by a multitude of sensors that generate large volumes of data. However, traditional condition monitoring techniques face several limitations: they rely heavily on high-quality, continuous sensor input, struggle with adaptability to new fault scenarios, require significant computational resources, and often provide limited decision support beyond fault detection. These constraints hinder their practical utility in dynamic and resource-constrained environments. This paper introduces a biomimetics-inspired framework for condition management, drawing on principles observed in natural systems to overcome the aforementioned challenges. Biomimetics, an emerging interdisciplinary field, has shown significant promise in bridging gaps between theoretical innovation and practical industrial application. However, its potential remains underutilized in maintenance decision-making systems. In response, our study proposes a biologically inspired methodology that parallels the human cognitive system, integrating multi-sensory data, adaptive learning, and energy-efficient sensing mechanisms to enhance fault diagnosis and decision-making. The core contributions of this research are fourfold: (1) adaptive intelligence through continuous learning that revises rules and cases over time; (2) multi-sensory integration, inspired by animal sensory systems, to improve diagnostic accuracy; (3) data augmentation techniques that address issues of incomplete or noisy input; and (4) the introduction of energy-efficient sensors and biomimetic optimization strategies suitable for IoT and edge devices. To demonstrate the practical applicability of our approach, we conducted empirical studies using vibration data for procedural analytics, validating the framework's effectiveness in real-world fault diagnosis. It serves as a functional roadmap, inviting broader discussion on the integration of biomimetics in maintenance engineering.

## 1 Introduction

Maintenance is extensively established as an essential business task and a critical element of asset management (de Jonge and Scarf, 2020). Maintenance actions can be distinguished into two categories, preventive and corrective, based on the incidence of failure. A preventive maintenance action is made before the failure of a unit; a corrective maintenance action is undertaken after failure. Preventive maintenance actions should be based on time, usage, or condition information. In maintenance engineering, decisions are often categorized into three levels: operational, tactical, and strategic. These decisions are made at different hierarchical levels within an organization and involve varying degrees of planning and execution. Operational decisions deal with the immediate tasks and activities required for ongoing maintenance, tactical decisions translate these into actionable plans, and strategic decisions set the overall direction for maintenance.

Condition-based maintenance (CBM) operates on the principle of “if it's not broken, don't fix it.” The equipment's status is continuously monitored by systems or machinery based on physical and process parameters to anticipate machine health, forecast faults, identify fault types, and predict machine failure timelines. The CBM system detects potential failures (PF) in their early stages. The evolution of CBM systems involves four levels: fault detection, fault identification, fault quantification, and fault prognosis (Kandukuri et al., 2016). The CBM implements a closed-loop maintenance strategy, wherein sensor data from equipment is gathered and utilized to inform planned maintenance decisions (Singh et al., 2024a). CBM aims to achieve optimum system reliability and safety while using the least amount of maintenance resources possible. The maintenance plan is based on the observation of degradation or damage that can be measured rather than a specific lifespan. The analysis can be split into diagnosis and prognosis based on the timeline. Diagnosis focuses on identifying the current condition using present observational data, while prognosis involves forecasting the future trajectory of the diagnosed fault, including its probability and consequences. In brief, the diagnosis is rooted in assessing the current condition evaluation, while prognosis relies on predicting future conditions (Ahmad and Kamaruddin, 2012). However, both have limitations in real industrial practice. For instance, the current condition evaluation method may not allow enough time for maintenance planning if the equipment condition has already reached or exceeded failure limits when evaluation results are updated. Combining diagnostics and prognostics enables the capture of the essence of the CBM concept. However, the reliability of future predictions remains uncertain. Short-term predictions are generally more reliable than long-term ones, rendering future condition prediction beneficial primarily for short-term planning scenarios where anticipation is necessary (Dhungana, 2025).

Reliability-centered maintenance recommends implementing CBM only when a sufficiently long potential failure interval exists. To extend this interval, it's essential to detect incipient faults early and utilize this information for immediate decision-making through procedural decision-making while considering safety and security concerns. Mean time before failure, mean time to failure, mean time to acknowledge, and mean time to repair are the most common terms to reflect time in the industrial CBM process. The time value of decision-making is a core principle of condition monitoring (CM). Correct decision-making in the current time has greater value than the same decision to be made in the future. Maintenance cost, risk, and downtime can be reduced only if a proper decision is made earlier. Despite advances in CM processes, several critical challenges in existing techniques remain unaddressed.

1. They are limited by their dependence on high-quality, continuous sensor data and are prone to accuracy issues when faced with missing, noisy, or incomplete inputs.
2. They have limited adaptability due to predefined thresholds and rule-based systems that struggle to adjust to changing operational environments or unknown fault conditions.
3. They are limited by high computational complexity, making advanced methods difficult to deploy in real-time or on edge devices.
4. They often have a narrow focus, being specialized for specific equipment or failures with limited predictive capability for early fault detection.
5. They often lack decision-making intelligence, identifying faults without providing sufficient guidance on optimal maintenance actions such as when, what, or how to maintain.

Biomimicry helps to solve modern-day technological problems using the solutions that were successfully evolved by bioorganisms over billions of years (King et al., 2015). Hence, it may be considered the most sustainable form of technology. Designing decision-making systems for CM is often intricate, relying on experience, intuition, and ongoing refinement. Bio-inspired decision-making frameworks have evolved, encompassing three primary types of decisions: procedural decisions, deliberate decisions, and argumentative decisions (Singh et al., 2024a). The procedural maintenance decision mainly depends on safety and security, whereas the deliberate decision focuses on cost. The deliberative maintenance decision is dependent on several factors, including downtime cost, frequency, and item reliability. Therefore, the balance between cost-cutting and CBM versus corrective maintenance may differ from one organization to another based on their assets and goals. However, the CBM system should overcome difficulties and find effective solutions to improve a situation or achieve a specific objective. These decisions hinge on the equipment's repairability and structure, whether it's single or multi-unit (Ahmad and Kamaruddin, 2012).

The integration of biomimicry into the cyber-physical system holds the potential for innovative approaches and the rapid adoption of new technologies, especially when profitability and sustainability are evident. Utilizing biomimicry as a decision-making framework for maintenance decisions helps maintenance engineers and other inspection professionals. Moreover, it introduces a realm of innovative possibilities for enhancing the decision-making process while prioritizing human wellbeing. By applying biomimicry principles, industries and maintenance professionals can explore a vast realm of innovative ideas to revolutionize the maintenance process, while simultaneously optimizing costs, time, and human wellbeing. Furthermore, beyond individual projects, biomimicry principles enable smarter decision-making that aligns actions with the natural environment. This holistic approach fosters sustainable practices and promotes a deeper connection between human activities and the natural world.

This work introduces a biomimicry-inspired decision-making methodology based on human cognition (sensation, perception, memory, learning, thinking, and problem-solving) to enhance industrial maintenance, featuring adaptive rule refinement, multi-sensory data integration, and energy-efficient processing for constrained environments. Key contributions include (1) demonstrating adaptive intelligence through ongoing case and rule updates, allowing systems to learn from experience, (2) empirical validation for rapid fault identification, and (3) advocating for energy-efficient sensors, context-conscious perception adaptation, leveraging data augmentation to address missing or degraded data, and biomimetic optimization algorithms ideal for IoT and edge applications.

To the best of our knowledge, this is the first study to present an extensive analogy between industrial CM and human cognitive processes, offering a novel path toward sustainable and intelligent industrial decision-making. The remainder of the paper is structured as follows: Section 2 offers an overview of biomimetic from present status and potential perspective focusing specifically on process biomimicry. Section 3 delves into the cognitive stages involved in decision-making. Section 4 details the structure and functionality of the bio-inspired decision-making methodology. Section 5 presents empirical studies along with a detailed discussion of the experimental results, evaluating the performance and effectiveness of the proposed methodology. Section 6 provides concluding remarks.

## 2 Biomimicry overview

### 2.1 Current status

Biomimicry is the practice of imitating nature's forms and processes to solve human problems. Benyus (1997), a biologist and leader in biomimicry, advocates for imitating nature to achieve a sustainable future. As Janine Benyus asserts, “Nature is inherently creative and has already solved many of the problems we encounter today.” Indeed, the natural world abounds with creativity and innovation. Biomimicry proponents emphasize that merely copying natural form is not enough (Bhushan, 2009). They propose a comprehensive approach encompassing three levels of mimicry: form, process, and ecosystem, as shown in Table 1. This entails considering the physical attributes, underlying mechanisms, and wider ecological context in order to effectively emulate nature.

**Table 1:** Three levels of biomimicry.

Form (Design or material) (Fu et al., 2020; Wegst et al., 2014)	Building interior architecture and design, Structure of material, Peak shape in high speed train Cements like corals, Dust repellent paint, etc.
Process (Methodlolgy) (Gao et al., 2019)	Sensing, survival, protection communicating technique Robotic locomotion, etc.
Ecosystem (Blanco et al., 2021)	The contextual fit, Camouflage for self-protection by octopus Nature recycles everything, Nature thrives with diversity etc.

The first level of design biomimicry has been using intensively in engineering design (Al-Obaidi et al., 2017; Fu et al., 2020; Chayaamor-Heil and Hannachi-Belkadi, 2017), material science (Wegst et al., 2014), soft robotics (Coyle et al., 2018), and sensor technology (Stroble et al., 2009). Nature serves as a rich source of time-tested material, structure, and patterns, which biomimicry aims to comprehend and apply to design challenges.

The second level of process biomimicry encloses intelligent behaviors and biological marvels, exemplified by insects and birds, which excel in complex tasks due to their adaptability, autonomous learning, and strength and efficiency. It involves drawing inspiration from natural processes to develop innovative industrial solutions to various condition monitoring challenges. This approach often leads to more sustainable and efficient solutions that mimic the resilience, adaptability, and effectiveness found in natural systems. Common examples of process biomimicry are robotic locomotion strategies (Gao et al., 2019), optimization, and metaheuristic searching algorithms (Dorigo et al., 2006; Daweri et al., 2020; Katoch et al., 2021) .

To date, biomimicry has primarily focused on the first two levels of design and process. However, to fully revolutionize our industries, we must replicate entire ecosystems, which pose greater challenges due to their complexity and multitude of factors. Mimicking entire systems enables the concept of “regenerative design,” which seeks to counter the ongoing degradation of ecosystems. This approach involves designing urban areas to restore and heal ecosystems, benefiting both nature and humanity (Blanco et al., 2021).

### 2.2 Potential perspective

Currently, biomimicry is relatively new in the field of CM. Despite the increasing availability of bio-inspired sensors and actuators, and the emergence of bioinspired analytics for data processing, decision-making frameworks that fully integrate biomimicry at a macro scale remain rare. Biomimicry will find wider application in maintenance engineering, particularly as a valuable long-term tool, as a means of fostering sustainable processes and optimizing deliberate decision-making.

#### 2.2.1 Energy-constrained and comprehensive sensing

Event cameras, inspired by biological vision systems, detect changes in brightness asynchronously at the pixel level and operate more energy-efficiently than traditional frame-based sensors. Biologically inspired sensor arrays emulate the distributed sensing capabilities of natural systems, enabling real-time, and energy-efficient data acquisition.

Event cameras represent a transformative advancement in condition monitoring. Unlike conventional cameras that capture frames at fixed intervals, event cameras asynchronously detect per-pixel brightness changes, generating a continuous stream of events that encode the time, location, and polarity of these changes (Gallego et al., 2022). This unique sensing mechanism offers several advantages like high temporal resolution, low latency, reduced motion blur, high dynamic range, and energy efficiency, making them suitable for CM in energy-constrained industrial settings.

In a big and complex system, a single sensor is incapable of accumulating enough data; by combining different sensor modalities, we can unlock synergistic benefits. A multi-modal sensor array combines different sensing modalities, such as temperature, force, and vibration, into a single unified system, allowing it to capture diverse data types simultaneously. This integration not only simplifies the system design, reducing complexity and cost, but also enhances the accuracy and depth of condition monitoring by correlating data from multiple sensors to provide more comprehensive and insightful analysis. For instance, a tactile sensor array equipped with thermal, force, and micro vibration modalities can detect mechanical forces, vibrations, and temperature variations (Lin et al., 2009). Overall, sensory arrays play a vital role in boosting the accuracy, reliability, and versatility of sensing systems across a wide range of applications. Comprehensive sensing approach can be implemented by consolidating all sensor inputs, enabling more informed, accurate, and timely insights in CM. for maintenance and fault detection.

#### 2.2.2 Perception enhancement via super resolution and data augmentation

Super-resolution algorithms enhance low-resolution images by predicting and adding missing details, akin to how the human brain reconstructs incomplete sensory information based on prior knowledge and experience. In condition monitoring, it can be applied to improve the clarity and accuracy of sensor data, such as vibration signals or images, allowing for more precise detection of anomalies or defects (He et al., 2024). By enhancing the resolution, super resolution helps uncover subtle patterns that may be overlooked in lower-quality data, thereby improving predictive maintenance and fault detection capabilities.

Organisms enhance survival by generating diverse variations to adapt to new environments; similarly, data augmentation creates synthetic data variations to improve machine learning model robustness across varying conditions. A model to guide the selection of appropriate data imputation techniques tailored to diverse measurement environments is presented in Dhungana P. et al. (2025); however, in certain scenarios, the collected data remains insufficient for making optimal decisions. Data augmentation techniques have proven effective in generating synthetic data that closely resembles and enhances the original training set (Semenoglou et al., 2023; Hou et al., 2022). Having many training datasets greatly enhances time series analytics.

#### 2.2.3 New evolutionary computation algorithms for optimization

New evolutionary computation algorithms are continually emerging by mimicking natural processes such as evolution, swarm intelligence, and cellular growth. These biomimicry-inspired methods enhance optimization by offering adaptive, scalable, and efficient solutions to complex real-world problems across various domains. In real-world engineering applications, numerous challenges arise that involve multiple viable solutions, which are commonly referred to as optimization problems. Within this context, the pursuit of identifying the optimal solution among the array of available choices characterizes the process known as optimization. The optimization problem is solved through four steps: parameter identification, recognition of constraints, consideration of objectives, and finally, based on the identified types of parameters, constraints, and number of objectives, a suitable optimizer should be chosen and employed to solve the problem (Saremi et al., 2017). The optimization algorithms usually fall into two types: gradient-based and heuristic. Metaheuristic algorithms are higher-level strategies for refining heuristic algorithms to guide the search process for finding optimum solutions. Metaheuristic algorithms, inspired by natural phenomena, do not rely on gradient information and can utilize a set of design points to find the optimum, making them highly robust.

Metaheuristic algorithms typically fall into two categories: single solution-based and population-based. In single solution-based algorithms, only one solution is processed during optimization, whereas in population-based algorithms, a set of solutions evolves in each iteration of the optimization process. They find optimal solutions for complex problems through random searches, drawing inspiration from natural phenomena, living organisms, physics, biology, human behavior, and other evolutionary concepts. Meta-heuristic optimization techniques have become very popular over the last two decades for four main reasons: simplicity, flexibility, derivation-free mechanism, and local optima avoidance. Meta-heuristics may be classified into four main classes: evolutionary, physics-based, swarm intelligence, and human-based algorithms (Kaveh and Mesgari, 2023).

Today, all evolutionary optimization algorithms are collectively known as evolutionary computation algorithms. Bio-inspired algorithms, a prominent field of artificial intelligence, has seen extensive study over recent decades. The two most widely used evolutionary algorithms are the genetic algorithm (GA), which draws inspiration from Darwin's principle of survival of the fittest (Katoch et al., 2021), and particle swarm optimization (PSO), which is based on a simplified social model (Dorigo et al., 2006). Genetic algorithms have effectively tackled diverse optimization tasks such as parameter tuning, scheduling, routing, and machine learning.

In the realm of open-source Python libraries for nature-inspired metaheuristic optimization algorithms, several general-purpose packages exist, including Opytimizer, NiaPy, EvoloPy3, DEAP4, and others (Faris et al., 2016). However, the most recent and comprehensive platform, Mealpy, stands out with its collection of over 160 classical and state-of-the-art metaheuristic algorithms (Van Thieu and Mirjalili, 2023). Developed through an analysis of existing libraries and validated via a case study discussion, Mealpy offers well-documented code, a user-friendly interface, and minimal dependencies.

In essence, biomimicry offers promising perspectives in CM by inspiring energy-efficient and comprehensive sensing strategies, particularly valuable in resource-constrained industrial environments. It also enables perception enhancement through techniques like super-resolution and biologically inspired data augmentation, which improve model robustness and handle challenges such as missing or incomplete sensor data. Additionally, bio-inspired evolutionary computation algorithms can drive more effective optimization in fault diagnosis and maintenance planning, contributing to smarter, more adaptive decision-making systems.

## 3 Cognitive stages in decision making

Understanding how humans think is difficult because countless mechanisms can lead to the same observation. However, recent research in various fields like economics, psychology, neuroscience, and linguistics has started combining different methods (Lieder and Griffiths, 2020). This includes adding cognitive limits to rational models, blending rational principles into how our minds work, and using optimization ideas to grasp how our brains represent things. The author showed that resource-rational models can explain how our minds perform complex cognitive tasks even though we sometimes act irrationally.

**Figure 1:**
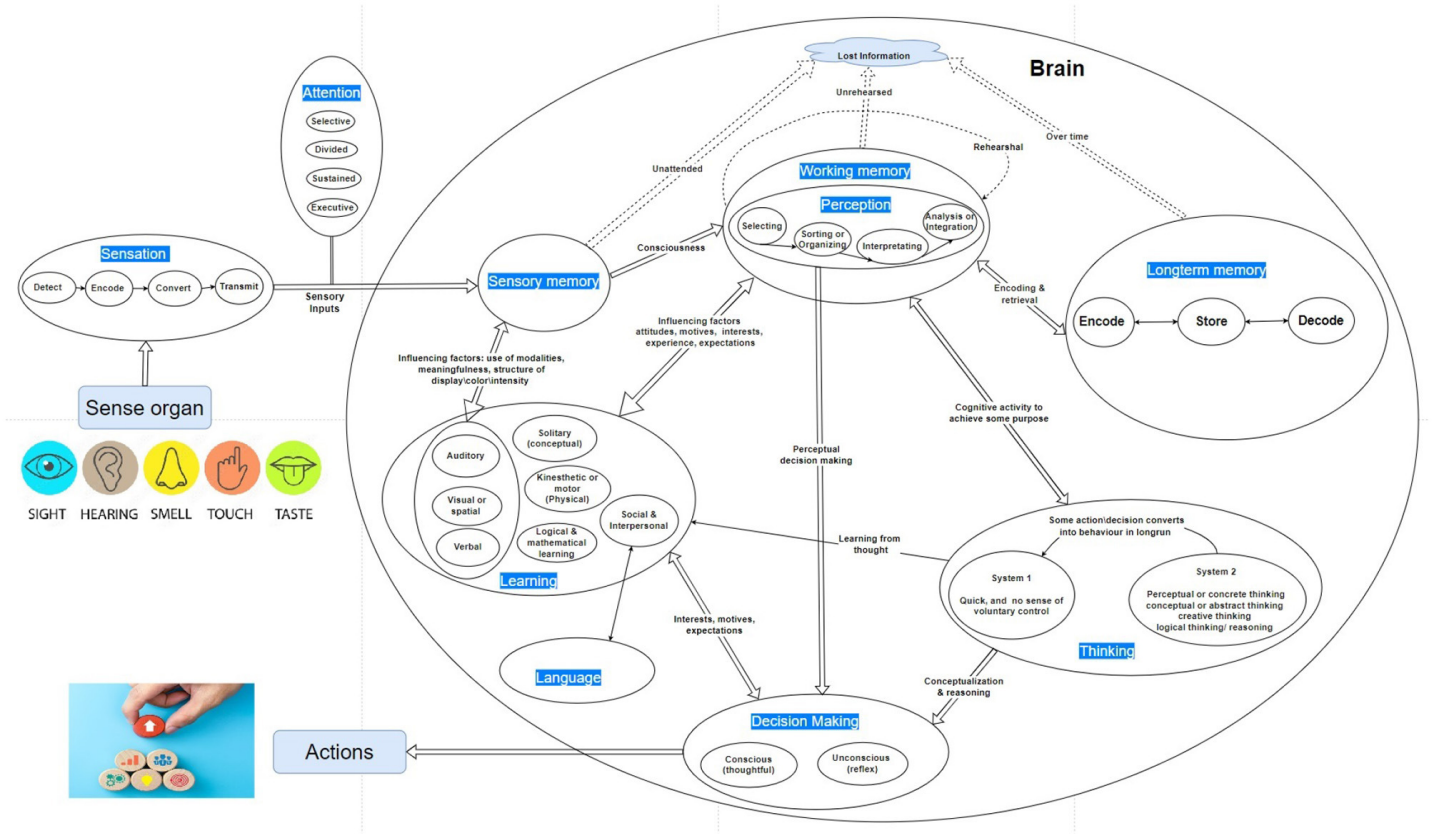
A model of human decision-making showing cognition stages from sensation to action.

According to the Oxford Learner Dictionary, cognition is the process by which knowledge and understanding are developed in the mind. It is a mental process of acquiring knowledge and understanding through thought, experience, and the senses. It encompasses many aspects of intellectual functions and processes, such as perception, attention, learning, memory, thinking, decision-making, and problem-solving. In other words, cognitive processes use existing knowledge from sensation, perception, and memory to discover new knowledge by learning, thinking, and decision-making. The complete cyclic diagram illustrating the cognition process from sensation to action is shown in Figure 1.

### 3.1 Sensing

To register changes in the environment, a biological organism has multiple senses. The sense organs convert changes in different environment variables into electrochemical signals and send them to the brain via sensory nerves. While human senses are limited to vision, hearing, touch, smell and taste, some animals employ a vast range of senses, like sonar in bats, hygroreceptor in Cockroach (Filingeri, 2015), electroreception in some ray-finned fishes, bumblebees, platypus (England and Robert, 2022; Newton et al., 2019), infrared radiation detection in snakes (Gracheva et al., 2010), or magnetic fields in frogs, snails, lobsters (Formicki et al., 2019; Wiltschko and Wiltschko, 2019).

Corresponding to the sense organs in animals, different types of sensors are used in condition monitoring. Some sensors may have equivalence in the animal world, while others may monitor specific parameters that have no equivalence in the animal world. Different sensors have their own structure and capability in terms of sample rate, resolution, accuracy, sensitivity, bandwidth, low power, etc. Selection of the instruments depends on the parameter that needs to be monitored. A summary detailing these sensory mechanisms, and their parallels in the industrial domain are presented in Table 2.

**Table 2:** Similarities between biological sensing and industrial condition monitoring.

**Animal**	**Sense organ**	**Feature**	**Bit transducer**	**Connection to nervous system**	**Condition monitoring**	**Industrial sensor**
Human	Eye (Sight)	Vision (Light intensity and visibility)	Photosensitive Rods cells and Cones cells on retina convert light into neural signal	Optic nerve	Visual inspection Laser interferometry	CC Camera, IR camera, computerized tomography
Human	Ear (Sound)	Hearing (Mechanical wave vibration)	Hair cell inside cochlea convert vibrations and knock into neural signal	Cochlear nerve	Acoustic monitoring	Acoustic microphone
Human	Nose (Smell)	Olfaction	Cilia in the epithelium convert smell signal into neural signal	Cranial nerves	Odor sensing Olfactory monitoring	Chemical/Gas, Smoke detector, antigen-based detection
Human	Tongue (Taste)	Gustation	Gustatory cells inside the taste buds convert tastants into neural signals	Vagus nerves	Potentiometric sensing, Concentration monitoring	Chemical/Antigen-based detection
Human	Skin (Touch)	Tactile perception	Tactile sensations (touch, pressure, heat, and vibration) into neural signal	Peripheral nerves	Thermography, Vibration monitoring, Touch inspection	Temperature/pressure contact vibration
Deep water fish	Skin (Electroreceptor)	Electric field	Electroreceptive ampullae of Lorenzini	Sensory nerve	Electric power distribution inspection	Electrical field strength sensor
Arthropods Mollusce and birds	Peak, skin (Magnetoreceptor)	Magnetic field	Electroreceptive ampullae of Lorenzini	Trigeminal nerve	Navigate using the earth's magnetic field	Compass/ Inclinometers
Cockroaches, Worms	Antenna (Hygrosensation)	Hygroreceptor	Hygroreceptive sensillum (moist and dry sensitive neural cells)	Sensory nerve	Air density monitoring	Humidity sensor
Snakes	Pit organ (Infrared radiation)	Infrared receptors	Radiation heating of Pit organ via molecular basis	Nerve fibers system	Thermal imaging	Infrared camera
Bats	Ears (Ultrasonic detection)	Ultrasound receptors Monitoring	Hair cell inside cochlea convert vibrations and knock into neural signal	Cochlear nerve	Ultrasonic Monitoring	Ultrasound sensor

### 3.2 Attention

Attention involves concentrating consciousness on a specific idea or object at a particular moment, excluding other stimuli. It is governed by a combination of voluntary processes, controlled by a central executive system, and involuntary processes, regulated by the attentional orienting system (Cowan, 1998). Humans process vast amounts of sensory information daily, but they filter out the familiar and focus on a small fraction. Thus attention prioritizes data with maximum information for conscious thought. Attention serves as an initial filter for human information processing, shaping our perception of environmental stimuli (Cowan, 2008). It can narrow to reduce irrelevant input or broaden to integrate parallel information streams.

In computational, attention is equivalent to filtering, reducing data size, choosing important features, and organizing how tasks are done. Attention mechanisms dynamically adjust the weights of input features, enabling models to focus on the most relevant information for both tasks fault diagnosis and failure prognostics. Incorporating attention into deep learning models enhances feature extraction by emphasizing critical patterns indicative of system degradation, thereby improving the accuracy and robustness of prognostic assessments in complex industrial environments.

### 3.3 Perception

Perception is the cognitive process of making sense of sensory stimuli, drawing on prior knowledge and environmental cues to construct a meaningful reality. It involves identifying, selecting, organizing, and interpreting sensory data. Recent research highlights the role of prior expectations in shaping sensory processing, either reducing or enhancing sensory representations before and after stimuli (de Lange et al., 2018).

Human actions, emotions, thoughts, and feelings are triggered by their perceptions, so different people may perceive the same environment differently. It is a subjective process and is like beauty that lies in the eyes of the beholder. The Gestalt principles in psychology are founded on the concept that the whole is greater than the sum of its parts (Todorovic, 2008). They elucidate how humans perceive and interpret visual data through principles such as similarity, proximity, closure, symmetry, and continuity. These principles of encoding shed light on how humans naturally perceive environmental observations. Similarly, perception in CM processes can be implemented by using raw sensor data of preprocessed features, to identify early-stage anomalies.

### 3.4 Memory

Human memory is filled with different information, knowledge, and skills and has the ability to revise the content. Every single content in memory is kept as a task and stored by encoding with model gained by observation or hardcoded at birth to represent knowledge (Hawkins, 2021). Different groups of nerve cells are responsible for different thoughts or perceptions and drift in and out of action. Different sensations (visual, auditory, tactile, olfactory, and gustation) experienced by the brain can exhibit distinct patterns of observation, and we can interpret these signals, resulting in a set of memories. Episodic memory is thought to depend on brain regions, such as the hippocampus and adjacent structures in the medial temporal lobe, along with connections to other brain regions responsible for memory consolidation and retrieval. Experience is the capacity to connect various tasks and knowledge, drawing upon past encounters and insights to navigate current situations effectively. As a person gains more experience, he can accumulate a greater number of models that enable him to approach and solve a wider range of problems and navigate various situations effectively. In the context of control, memory is classified into sensory, working, and long-term memory, with information flowing from sensory receptors to working memory and potentially being stored in long-term memory (Atkinson and Shiffrin, 1971). Effective memory strategies such as encoding, storage, retrieval, and repetition are necessary to transfer information from working memory into long-term memory and *vice versa*.

Likewise, in CM, past instances serve as memory, which contains the specific data in the form text, image, video, programs, and algorithms in the form of files. The file format serves as a standardized method to encode, store, and decode as per analysis requirements. The content within these files varies and is determined based on type of sensor, analytical procedure, and system goals.

### 3.5 Learning

Learning is a process of acquiring knowledge, skills, attitudes, or behaviors through experiences, study, instruction, or observation. It involves a change in an individual's mental structures or behavior as a result of these experiences. It is explained by three theories: behaviorism, which emphasizes stimulus-response relationships (Kevin, 2018a); cognitive theory, which focuses on internal mental processes like information processing (Kevin, 2018b); and constructivism, which views learning as individual knowledge construction through interaction with the environment, involving experimentation, concept organization, personal meaning-making, and synthesis (Kevin, 2018c). In one sentence, the relationship between learning and decision making is symbiotic, with learning provide better insights for decision making.

These learning approaches are crucial in CM where vast and complex data must be interpreted accurately to detect faults. While machine learning and deep learning enable the system to learn patterns from raw sensor data, transfer learning significantly enhances performance by leveraging pre-learned knowledge from similar machines or conditions, reducing training time and improving diagnostic accuracy in scenarios with limited labeled data. Imaginative AI, transfer learning, and context-aware analytics collectively enhance deliberative decision-making by enabling creative solution generation, leveraging prior knowledge for new problem contexts, and integrating environmental and operational factors to produce insightful, adaptive, and context-sensitive maintenance strategies aligned with human-like learning and reasoning processes (Emmanouilidis et al., 2019). Bio-inspired computation, a prominent field of artificial intelligence, has seen extensive study over recent decades (Del Ser et al., 2019). Numerous innovative approaches have demonstrated the effectiveness of adopting various bio-inspired behaviors and characteristics to achieve near-optimal performance across a diverse array of complex academic and real-world problems to address complex modeling, simulation, and optimization problems.

### 3.6 Thinking

Thinking is the action of using one's mind to produce thoughts and concepts to solve problems, make decisions, form judgments, and generate new insights. It is used to reason, analyse, imagine, conceptualize, and make sense of information and experiences (Minda, 2020). For decades, psychologists have been deeply intrigued by two modes of thinking (Kahneman, 2011). System 1 functions automatically and rapidly, requiring minimal effort and lacking a sense of voluntary control, while System 2 directs attention to demanding mental activities, including complex computations.

### 3.7 Decision making

Decision making refers to the process of selecting a course of action or making a choice among available alternatives (Klapproth, 2008). It involves evaluating different options, considering relevant factors, and selecting the most suitable choice based on personal preferences, goals, and values. In neuroscience, the authors explain three types of information processing systems for decision-making (Van Der Meer et al., 2012). The Pavlovian system is recognized for promoting automatic and instinctive behaviors. The Habit-Based system involves transitioning a conscious decision into habit formation. Effective decision making requires critical thinking, problem-solving skills, the ability to analyse information, and a consideration of both short-term and long-term consequences. It also involves managing uncertainty and making choices in situations where complete information may not be available.

Cognitive activities like learning, thinking, and decision-making can be partially analogous to data analysis in the cyber domain. Data analytics connects data into reality, considering the forces behind the data. It's the art of interpreting reality using data, not merely reflecting data from various perspectives. However, human cognition is notably more intricate and adaptable than current computer algorithms. Humans adeptly manage ambiguity, context, emotions, and ethical factors, which pose challenges for machines to fully replicate. Recent advancements in artificial intelligence and machine learning aim to narrow the gap between human cognition and computational processes (Fridman et al., 2019).

## 4 Bio-inspired methodology for decision making

Cognitive modeling is vital for understanding the human mind, serving three main purposes. First, it allows for the precise testing of psychological concepts through computer simulations, pinpointing any explanatory gaps. Second, it facilitates the exchange of insights between human and artificial intelligence development. Third, modeling empirical phenomena helps infer the underlying psychological mechanisms essential for predicting human behavior in new scenarios.

A typical decision-making system follows the Sense–Think–Act model (Côté, 2019), where an agent gathers information from its environment (Sense), processes this information to make decisions (Think), takes appropriate actions (Act), and repeats these steps to achieve autonomy. A typical approach to condition-based decision-making unfolds as follows: (1) Sensors are deployed on equipment or structures to collect a substantial amount of data. (2) Data from sensors are analyzed; the quality and quantity of gathered data influence the maintenance decision. (3) An algorithm detects potential damage, pinpoints its location, and assesses structural health and performance. (4) The responsible manager decides whether to proceed with repairs. Essentially, decision-making is perceived as a step occurring once the manager comprehends the equipment's condition through the monitoring system. The increasing prevalence of terms like “smart equipment” or “intelligent monitoring” within our community underscores the notion that decision-making is essentially an outcome of condition monitoring.

The data–information–knowledge–wisdom hierarchy (DIKW) hierarchy, also known as the Knowledge Hierarchy or Knowledge Pyramid, is a widely acknowledged and fundamental model in information and knowledge literature (Rowley, 2007; Baskarada and Koronios, 2013). Conventionally, information is conceptualized in relation to data, knowledge in relation to information, and wisdom in relation to knowledge. However, there is less agreement on how the processes transforming elements lower in the hierarchy into those above them are described, resulting in a lack of definitional clarity.

Based on the data science domain in condition management, we redefine the terms of data, information knowledge, and wisdom in our case. Data is raw information about real or simulated entities, lacking inherent meaning. Data items serve as elementary and recorded descriptions of things, events, activities, and transactions. They comprise discrete, objective facts or observations that are unorganized and unprocessed, devoid of specific meaning, which comprises numerical values, textual statements, or other descriptive details concerning an object or concept. Information is meaningful data, often referred to as interpreted data and adds value to the understanding of a subject for humans. Knowledge is organized and processed meaningful data conveying understanding, experience, accumulated learning, and expertise relevant to a current problem or activity. In other words, knowledge contains profound relationships between different pieces of information and is integrated with opinion, skills, and experience, forming an asset for decision-making. Wisdom represents the highest level of abstraction, incorporating vision, foresight, and the capability to perceive beyond immediate circumstances. Wisdom involves the practical and critical application of understanding in diverse situations, guided by ethical judgment aligned with an individual's belief system. It is the accumulation of knowledge, enabling the application of concepts across different domains. Wisdom makes the best application of knowledge in a meaningful and ethical way.

Taking a computer science perspective, we define knowledge as validated information concerning relationships between entities in specific contexts. In the context of machine learning, a crucial element of knowledge is its formalization (Vonrueden et al., 2021). The level of formalization is contingent on factors such as written representation, structure, and the formality of the language used (e.g., mathematical formulas, logical rules, knowledge graphs, etc.). Greater formal representation enhances integration into analytical processes.

**Figure 2:**
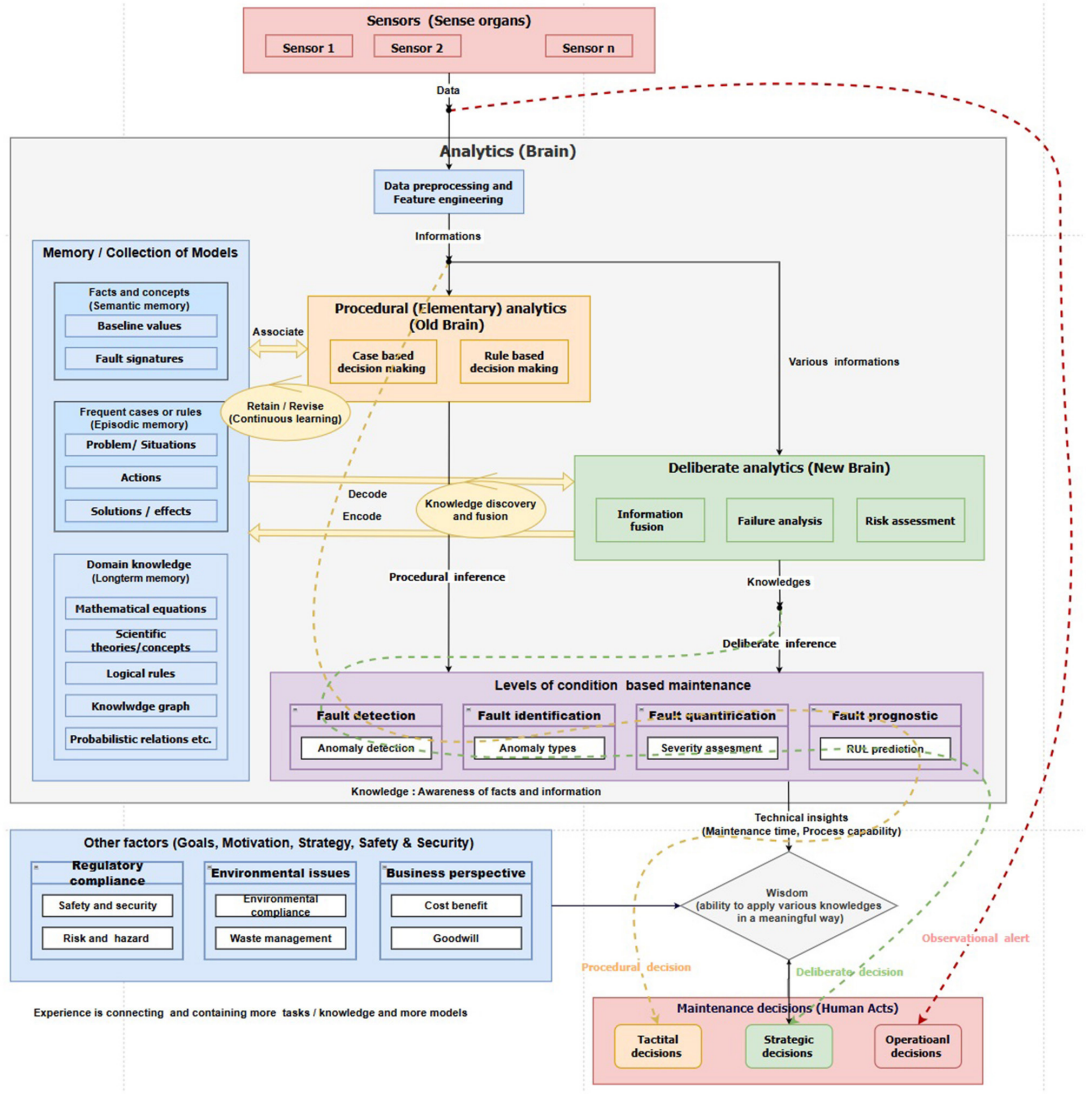
Functional block diagram of decision-making methodology for condition monitoring.

A structure designed for an intelligent decision support system centered around human needs and a robust decision depends on both the amount of data available and the effort invested in the decision-making process. In this framework, maintenance decisions occur at three different stages of data processing in the DIKW hierarchy. Decision-making directly from raw data is termed reflex decision, while decisions made from interpreted data (information) are procedural decisions. Finally, decisions derived from the meaningful relationship of interpreted data (knowledge) are termed deliberate decisions, as illustrated in Table 3 and Figure 2.

**Table 3:** Depending on where the data is extracted from within the DIKW hierarchy, different types of decision-making emerge.

Sensors ————————————— Data (number, text, image, audio, video etc.)
Data + meaning ——————————————————— Information (features)
Information + context (relationship with circumstances) ———————————- ———————————————————————- Knowledge (Risk, Reliability)
Knowledge + insight (consequences and likelihood) —————————————– ———————————————————————————– Wisdom (Decision)
Data ——————————————————————————– Reflex Decision
Information—————————————————————– Procedural Decision
Knowledge———————— Deliberate Decision (Wisdom and advance analytics)

### 4.1 Reflex decision

Momentary valence in human cognition refers to the immediate state of an individual at a specific point in time. It represents the subjective experience of the pleasantness or unpleasantness of an emotion felt in the present moment. Habitual behavior differs from procedural behaviors and demands considerable information processing (attention to environmental cues, logical checks for the correct pattern of conditions), while habits are automatic responses that need minimal information processing (Verplanken and Vemund Rudi, 2014). From the inspiration from the momentary valence and reflex snaps in human cognition, a reflex decision is presented.

**Figure 3:**
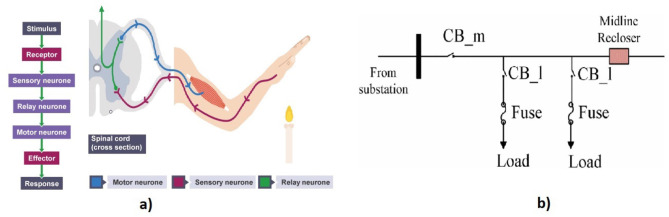
**(a)** Reflexive decision-making in human cognition (BBC Bitesize, n.d.). **(b)** Layout of distribution feeder adapted from Kumari and Naick (2022), where the instant activation of a circuit breaker in power distribution systems forms phasor measurement unit sensor data.

A reflex synapse is an automatic and nearly instantaneous movement in response to a stimulus and acts as an impulse before that impulse reaches the brain. A reflex is made possible by reflex arcs, which consist of sensory neurons, relay neurons, and motor neurons, and can act on an impulse before that impulse reaches the brain. An example of execution of reflex decision by pulling a hand away candle is shown in Figure 3a. The process begins as the receptor detects the stimulus, transmitting electrical impulses through the sensory neuron to the CNS (specifically, the spinal cord). At synapses between neurons, chemicals facilitate the passage of electrical impulses along the relay neuron and, subsequently, the motor neuron. Finally, the impulse reaches the effector muscle, causing actions like narrowing the eye in response to bright light or contracting muscles to move the hand away from pain.

Similarly, an emergency decision refers to an immediate activity required to handle seriously threatening situations to prevent or mitigate dangerous situations like burning, breaking, and other destruction. The decision region converts raw data into a reflex decision based on an inherent process. Regardless of the threshold level of an alarm system, machinery has various protective circuits, fuses, and circuit breakers. Thus, the immediate decision taken from sensor data to protect the equipment is categorized as a reflex decision in this framework.

For example, in power distribution systems, reliability is hampered because of a lack of adequate responses from the protective equipment. The system's functionality may be compromised when insufficient coordination between the fuse and the circuit breaker exists. To address this issue, a fuse-saving scheme was developed by leveraging the coordination between the fuse and circuit breaker using the Petri net model in Kumari and Naick (2022). Figure 3b illustrates the correlation between reflexive decision-making in human cognition and the immediate triggering of a circuit breaker. This decision aims to minimize the cost of fuse repair by preventing fuse burnout during a temporary fault.

### 4.2 Procedural analytics

Humans often rely on simplified decision-making processes and heuristics due to their cognitive limitations and the constraints of time and resources, as in the old brain. Although a bounded rationality concept was introduced by economists, it is highly applicable in procedural decision-making (Lieder and Griffiths, 2020). In CBM, sensors may collect noisy data and contain insufficient information, the decision support frameworks may have limited analytical algorithms and computation resources; however, the framework must provide satisfactory judgements. Procedural decision-making relies on prior cases and generalized rules. It might be useful in ensuring fair and consistent decisions, but sometimes, it may not be sufficient to accommodate unique or unexpected situations in decision-making. The procedural analysis aimed to investigate a simple, quick, and easy way of making decisions using experience and immediate goals. These decisions, while not optimal, are good enough to run the process equipment safely. This means that at the initial stages of the functioning of equipment, when there is no historical data to process and analyze, the equipment should be able to run safely, even sub-optimally. Once the basic information is gathered, the procedural analytics either utilizes association with prior cases or employs generalized rules from expert domain knowledge to reach prompt decisions.

#### 4.2.1 Case-based decision making

A case represents a condition of equipment and can be formulated in various ways, ranging from straightforward (linear) to intricate hierarchical structures. Typically, a case encompasses both a problem description and its corresponding solution. Case-based decision theory (CBDT) can be easily incorporated into this module for decision-making. Cases are stored as lookup tables in traditional discrete episodic memory models; however, in the cases of a continuous domain, where a state is never visited twice, previous episodic methods fail to aggregate experience across trajectories efficiently. In those situations, generalizable episodic memory is the practical solution for decision-making.

CBDT eliminates the need for an explicit domain model, transforming elicitation into the collection of case histories (Watson and Marir, 1994). Implementation involves identifying key features describing a case, a simpler task than creating an explicit model. Through the application of database techniques, CBD efficiently manages large volumes of information. CBD systems can also enhance maintenance by learning and acquiring new knowledge from cases.

**Figure 4:**
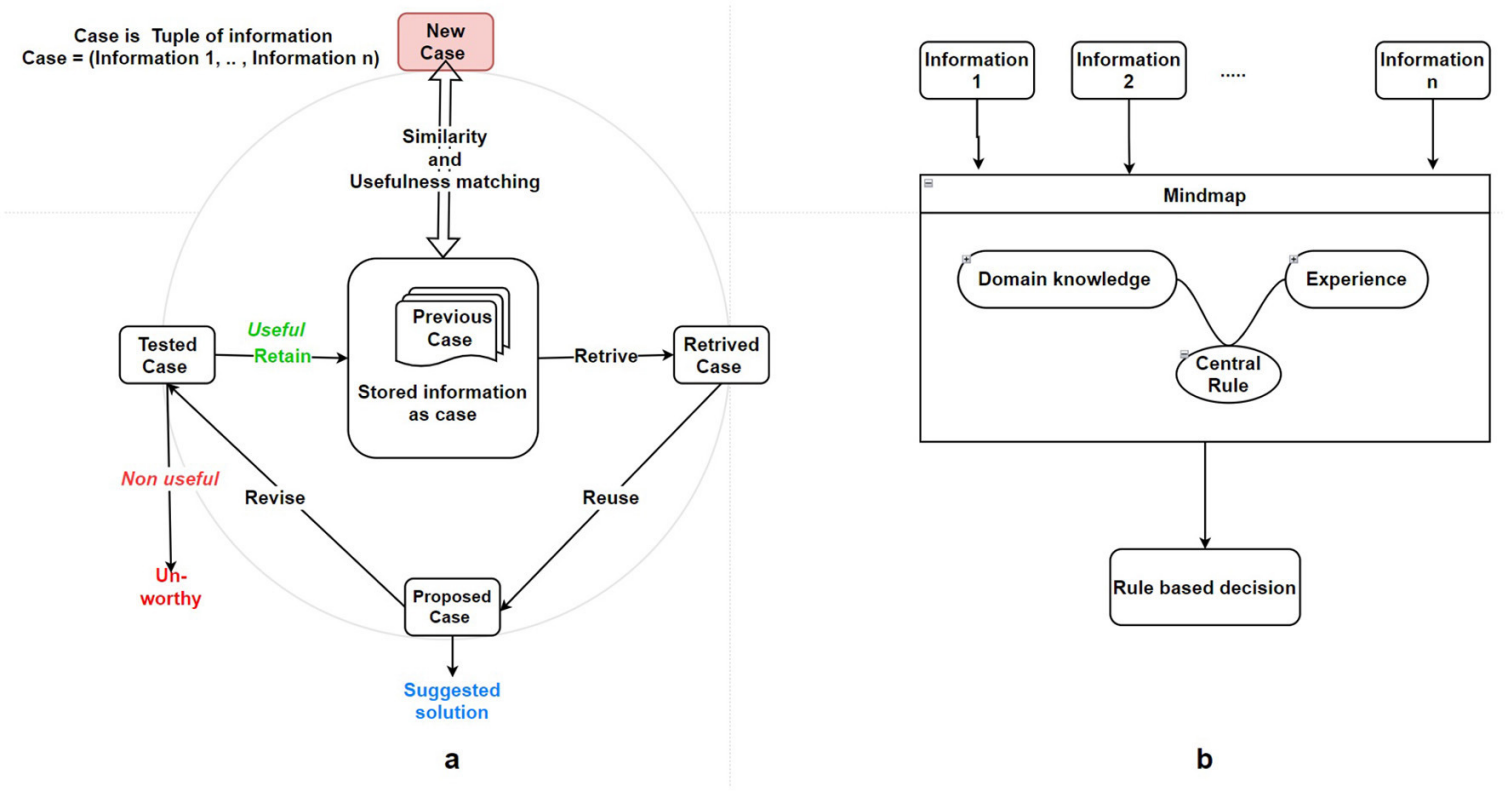
**(a)** Case-based decision-making model in condition management adapted from Agnar and Plaza (1994). **(b)** Decision rules interpretable machine learning.

The case-based decision-making process can be summarized succinctly in four steps: Retrieve, Reuse, Revise, and Retain, as shown in Figure 4a. Retrieve: When confronted with a new problem, recall the most similar cases. Reuse: Apply the retrieved cases to solve the problem at hand. Revise: Evaluate the proposed solution (continuous learning); if deemed inadequate, adjust to a better fit for the current problem or meet specific requirements. Retain: Store the proposed solution in memory as a newly solved case for use in future problems.

New information is modeled primarily by adding cases to memory, which stores only real occurrences. Each case informs solely about the chosen action within it, with the evaluation based on the actual outcome derived from that specific case. Case retrieval is based on two key criteria: usefulness and similarity. The equivalence term assumes the existence of a case utility function and a similarity function. Thus, the most similar and useful cases are retrieved, and based on that, each possible decision is evaluated.

Humans continuously learn and adapt by registering environmental changes, updating their mental models accordingly, and learning from mistakes to refine predictions based on stored models, rectifying errors when predictions fail to match actual changes. Similarly, case revision enables updates to existing models, while new information obtained is retained in memory for future scenarios and additions of new models; thus, continuous learning mirrors retraining a model in machine learning, which ensures consistent accuracy with the latest data, albeit with the crucial need to balance cost and accuracy when defining an optimal retraining schedule. Reusable cases are stored in a case base for future use in decision-making.

#### 4.2.2 Rule-based decision making

In rule-based decision-making systems, decisions are formulated as logical rules that incorporate elements of probability and fuzziness to handle uncertainty and ambiguity. Each rule comprises multiple components, with specific conditions guiding the selection of alternatives based on environmental cues. The Rule-Based Decision Field Theory (RBDFT) extends this framework by modeling dynamic attention shifts among competing options, allowing for adaptive decision-making in response to changing contexts (Johnson and Busemeyer, 2014). Fuzzy inference systems further enhance this approach by employing “if-then” rules to represent and reason about imprecise knowledge. Each fuzzy rule consists of an antecedent (“if” part) and a consequent (“then” part), facilitating decision-making under uncertainty. These systems are particularly effective in applications such as fault diagnosis and failure prognostics, where they enable the identification of critical patterns and support procedural decision-making processes.

Domain knowledge is important for creating rules. In the implementation part, a primary challenge arises in extracting knowledge and translating it into a set of rules as shown in Figure 4b. Consequently, knowledge extraction from experts stands out as a key issue in rule-based systems. Another challenge lies in the disparity between the complexity of the actual application domain for decision-making and the straightforward structure of IF-THEN rules.

Frequent repetition of a task can result in the formation of routines, which may replace more deliberate processes in influencing behavior. That means, repeated experiences lead to the development of simple rules or attribute values that inform successful decisions. The decision maker relies on either attribute values or rule-based guidance, with preferences evolving over time through the accumulation of this information. Similarly, when the system comprises numerous similar cases, it is natural to derive rules from repetated cases (Avdeenko and Makarova, 2017). As a simple example, a mere counter can quantify the experience.

In case-based decision-making, explicit rules are not established. However, when the system encounters recurring issues, it is common practice to evaluate an action based on its average past performance, rather than relying solely on a straightforward summation. It emphasizes the generalization of cases, encouraging the creation of basic, simple, and quick rules (Avdeenko and Makarova, 2017). Generalizable episodic memory, which encompasses the ability to abstract and generalize past cases, can serve as an intermediate step for rule-making. By extracting common patterns and regularities from past cases, rules can be formulated to guide decision-making. In essence, the generalization of cases paves the way for the creation of rules to guide decision-making in a variety of contexts. These explicit rules can be cleaned, improved, and used more effectively.

To a large extent, case-based decision-making and rule-based decision-making are not competing methodologies; they represent different approaches to cognitive behaviors. Rather than determining which approach is more appropriate for decision-making, it should be decided by system experts. In general, rule-based decision-making is simple and promising. However, if the system is new and there is limited history of failure cases, case-based decision-making might be the default choice. Both methods are equally simple and quick in terms of computation. Furthermore, the specific methods presented in these frameworks may not provide the same predictions given the same observations. Hence, we believe that there is room for both strategies.

### 4.3 Deliberate analytics

Deliberative analytics carefully consider various alternatives, weighing pros and cons, and making decisions based on reasoning. It deals with data, identify patterns, and interpret information to gain insights into potential threats or vulnerabilities. Information fusion, failure analysis, risk assessment, and optimization form the common elements of deliberate analytics.

#### 4.3.1 Information fusion

Information fusion in deliberate analytics combines information from multiple sources (sensors, memory, algorithms, or methodologies) to improve the quality of knowledges and has gained significant attention in condition monitoring. This fusion may involve integrating information to create a more comprehensive understanding of the situation. Experimental verification showed that collaborative fault diagnosis gives more reliable results by the integration of multisensory data and fusion of maintenance strategies (Shao et al., 2021). Knowledge-level sensory fusion integrates sensor signals' reported results based on voting fusion rules to offer collaborative inference.

#### 4.3.2 Failure analysis

Failure analysis is a systematic process of investigating the causes and consequences of a failure. It identifies the root causes of the failure, understands the mechanisms involved, and provides insights to prevent similar failures in the future; it can be conducted through various methods and tools, including Failure Mode and Effects Analysis (FMEA) (Mikulak et al., 2017), Fault Tree Analysis (FTA) (Sharma and Sharma, 2010), Root Cause Analysis (RCA) (Tuninetti et al., 2021), Reliability Block Diagram (RBD), 5 Whys, Probabilistic Risk Assessment (PRA), etc. Each of these approaches possesses unique capabilities and limitations, and the selection of a particular method depends on the specific requirements and objectives of the system at hand.

The three-level hierarchy in causal reasoning for failure analysis is outlined in Table 4. It addresses the activity and characteristic questions that can be answered at each level, along with additional examples related to condition monitoring analytics. The first level of the causal hierarchy is associative. It relies on purely statistical relationships defined by the raw data. The second level is interventional. It goes beyond observational raw data and focuses on changes being tested. Randomized controlled trials (RCTs) are widely regarded as the gold standard for assessing the effectiveness of interventions. They are valued for their ability to minimize bias and establish causal relationships between interventions and outcomes. Lastly, the third level involves Counterfactuals, which have been given computer-friendly interpretations in recent decades. A common question in this category is: “What if I had made a different choice?” This requires looking back and considering alternative actions. It draws upon scientific thinking, legal and moral reasoning (Pearl, 2019). In brief, interventional questions need more than just looking at statistics; similarly, counterfactual questions need more than intervention.

**Table 4:** The causal hierarchy adapted from Pearl (2019), the last four rows are newly added specially for condition monitoring.

**Concept**	**Association**	**Intervention**	**Counterfactual**
Level (symbol)	*p*(*y*|*x*)	*p*(*y*|*do*(*x*))	*p*(*yx*|*x*_0_, *y*_0_)
Typical activity	Seeing	Doing, intervening	Imagining, retrospection
Typical question	How does *x* change my belief in *y*?	What if I do *x*?	Would *y* have happened instead of *y*′, if I had done *x* instead of *x*′?
Example	What information does sensory data provide about the degradation score?	What if we change operation variable, will the degradation score be improve?	Would equipment still be functioning if the fire hadn't occurred.
Nature of analysis	It involves passive observation of naturally occurring events.	It actively involves manipulating variables in controlled experiments.	It compares actual outcomes with hypothetical scenarios.
Control	It lacks researcher control over variables.	It allows for researcher control and manipulation.	It relies on hypothetical scenarios to estimate causal effects.
Purpose	Lacking controls over variables make it challenging to establish causation.	It is focused on establishing causation and determining the impact of specific interventions.	It is concerned with estimating causal effects by exploring alternative scenarios.

Relying solely on observations for causal inference isn't enough; conducting intervention gives deeper understanding. Causal Bayesian networks are utilized for predicting intervention effects and formulating the corresponding transition probabilities in Lee et al. (2024). Predictive analysis entails forecasting future events by leveraging historical data and identifying patterns. Markov Decision Process (MDP) models have been widely employed in predictive analytics within stochastic domains, including applications in condition monitoring. An MDP model consists of states, actions, transition probabilities, and rewards.

Imagination is central to human cognition, and it involves in decision-making (Paul, 2014). Blomkvist (2022), claims that imagination is intentionally controlled by employing the reality constraint and change constraint. Imagination involves mentally going beyond time, place, and situations. The paper discusses the challenge of designing imagination machines, explores connections between current research and imagination, and explains how automating imagination can greatly progress AI (Mahadevan, 2018).

Creativity entails generating novel ideas, particularly when encountering complex challenges, and thinking outside the box. This creative thinking empowers decision-makers to explore unconventional possibilities, ultimately fostering innovative solutions and yielding superior outcomes. Similarly, within the realm of computer science analytics, when learning from observations and intervention proves inadequate for decision-making, there arises a necessity for counterfactual analytics, serving as the foundation for causal inference (Höfler, 2005). Creative AI can produce realistic video footage, images, graphics, and simulation data using techniques such as GANs, VAEs, and counterfactual simulations (de Vries, 2020). The simulated data can used for data augmentation to make more optimal decision.

Counterfactual analysis serves as the cornerstone of causal inference, especially in failure analysis, particularly for identifying root causes. Counterfactual decision theory (CDT) suggests that you should assess an action based on the outcomes that would probably happen if you were to do it (Hedden, 2023). Causation and counterfactuals are frequently associated, but not invariably. In instances such as overdetermination, constitution, and determinism, where they diverge, CDT provides accurate verdicts, supported by valid reasoning compared to ausal decision theory. Counterfactual causality was the primary concept driving the development of randomized experiments to generate simulated data. Counterfactual simulations are needed to explain causal judgements, and just hypothetical simulation isn't enough to predict causal judgements (Gerstenberg, 2022).

#### 4.3.3 Risk assessment

Risk pertains to situations where probabilities are known, while “uncertainty” refers to situations where states are defined naturally or can be easily constructed, but probabilities are not specified (Gilboa and Schmeidler, 2001). Risk can be broadly characterized as the likelihood of potential loss of valuable assets, while vulnerability encompasses the characteristics and conditions that may contribute to heightened risk and, consequently, potential losses (Escobar Wolf et al., 2018). Risk assessment is a well-established discipline where the systematic execution of assessments guides analysts in identifying potential hazards or threats, analyzing their origins and consequences, and presenting the risk, usually with quantitative precision and a proper representation of uncertainties (Zio, 2018). It has been the primary approach for ensuring safety in the design and operation of industrial systems.

The risk assessment module typically comprises three key stages: risk identification, risk analysis, and risk evaluation. In the first stage, potential risks are identified. The second stage involves a detailed analysis of these identified risks, assessing their causes, potential consequences, and likelihood. Finally, in the third stage, the analyzed risks are evaluated to prioritize and determine their significance, guiding subsequent decision-making and risk management actions. Risk assessment dependability modeling methods provide insights into various dependability modeling approaches from fault trees, Bayesian networks, stochastic Petri-nets, and so on, and how uncertainty is handled (Chemweno et al., 2018). However, the success of these strategies depends on adequately considering asset failure dependencies during the risk assessment process. In short, risk assessment helps organizations prioritize and allocate resources to address risks effectively.

#### 4.3.4 Knowledge discovery and fusion

Initially, early knowledge fusion predominantly relied on conventional data fusion techniques. Its primary focus was on validating data authenticity, ensuring the reliability of information sources, resolving numerical conflicts between diverse data sources, and deducing the inherent real value. This approach placed particular emphasis on the data level. Contrasting with data fusion, knowledge fusion diverges in its fundamental objective. It revolves around the exploration of methods to amalgamate descriptive information concerning a singular entity or concept from various sources (Zhao et al., 2020). Knowledge graphs, fuzzy set theory, Dempster-Shafer evidence, and Bayesian inference are common theories used for knowledge discovery and decision-making.

The Knowledge Graph (KG) is recognized as an evolving semantic infrastructure for big data analysis and knowledge discovery (Ristoski and Paulheim, 2016). It serves as a structural semantic knowledge base, employing a graph-based representation method to symbolically describe real-world concepts and their relationships. Its efficiency in managing and representing massive, multi-source data has led to its application in equipment condition management (Qiu et al., 2022). This structured representation gives relationships, identifies patterns, and makes informed decisions based on a comprehensive understanding of the information for decision-making.

Petri nets serve as graphical and formal modeling tools for fault analysis known for their expandability and portability (Peterson, 1981). They can be seamlessly converted from fault trees, offering robust logical description capabilities. Murata (1989) demonstrates the transformation of various intricate fault tree relationships into Petri nets, showing their efficiency compared to fault trees. A formally defined as a directed bipartite graph using Petri net of a six-tuple was presented for failure analysis in Sharma and Sharma (2010).

Fuzzy set theory is effective when dealing with both inaccurate and uncertain information. It allows for the representation of uncertainty using degrees of membership, making it suitable for situations where precise data is difficult to obtain. Its efficacy hinges on the precise modeling of membership functions, a task that may demand domain expertise. It may not be as well-suited for problems with highly structured and precise data.

The Dempster-Shafer Theory of Evidence (DSTE) is based on two principles: (i) assessing degrees of belief for subjective probability estimates, and (ii) combining these degrees of belief within a probabilistic framework (Shafer and Logan, 1987). It offers an effective method for integrating information from various sources, qualitative and quantitative alike, using Dempster's combination rules and advantages to deal in conflicting evidence (Lin et al., 2015). Moreover, it can handle situations where information is incomplete or when there is uncertainty about the reliability of different sources, but its implementation is complex and computationally intensive.

Bayesian theory is well-suited for updating beliefs and probabilities based on new evidence. It is particularly effective when dealing with well-defined probabilistic models and when there is a need to incorporate prior knowledge into decision-making. It assumes a clear specification of prior probabilities and likelihoods, which may be challenging in cases with limited historical data or when uncertainties are high. In knowledge discovery, the choice between these theories could depend on factors such as the type and quality of available data, the level of uncertainty in the system, and the modeling requirements. Additionally, some applications may benefit from a combination of these approaches or from integrating them into a hybrid framework to leverage their respective strengths.

### 4.4 Level of condition based maintenance

Condition monitoring (CM) systems evolve through four levels of capability: detection, diagnosis, quantification, and prognosis (Kandukuri et al., 2016). At the basic level, CM can detect the presence of faults, progressing to diagnosing the type of fault, then quantifying its severity, and ultimately predicting future failure timelines.

#### 4.4.1 Fault detection

Fault detection level has the capability to detect faults and differentiate between healthy and faulty states. The fault is identified either based on baseline values stored in memory or by detecting anomalies from observations. Fault detection in condition monitoring can be modeled after the trigger drawn from attention. Just as attention concentrates on relevant senses in human cognition. The fault detection module can discern incipient faults, distinguishing between a healthy state and a degradation stage. Machine learning model was used to identify the patterns and anomalies in Dhungana et al. (2024).

#### 4.4.2 Fault identification

Fault identification possesses the capability to determine the type of fault that has occurred and pinpoint the location of the fault within the equipment. Identification relies on recognizing prior fault data stored in memory. In our previous work, we applied a signature-based initial fault identification approach as evidence of the effectiveness of this example (Singh et al., 2024b).

#### 4.4.3 Fault quantification

Fault quantification level captures the magnitude of incipient faults. At the early fault detection stage, it may be challenging to measure the fault size precisely. However, certain statistical features can provide an approximate indication of the fault magnitude based on the distribution of measurements. Capability index, six Sigma are the common parameters to quantify fault severity because reflect the degree of the process variation with respect to the specific limits (Gupta et al., 2018).

#### 4.4.4 Fault prognosis

Fault prognosis aims to forecast the progression toward functional failure. In failure prognostic problems, fault quantification information can be utilized to model the rate of degradation and predict the remaining useful life (Dhungana et al., 2025b). Based on the degradation rate, the remaining useful life is predicted to set maintenance time.

### 4.5 Memory

#### 4.5.1 Baseline references and signatures

The baseline represents the operational value when the equipment normally operates. Detecting fault conditions as early as possible, with a specified degree of confidence, while minimizing false alarms is essential. Therefore, the availability of historical data is assumed to define an appropriate baseline. Baseline values are subjective and established based on best practices and experience, in line with system requirements. In our previous work of initial fault finding, the average of four experimental readings are employed as baseline value (Singh et al., 2024b). Signatures are unique patterns of data or information utilized for potential matching with historical data for classification and clustering. The instantaneous signature of the observed data is compared with a database of fault signatures stored in memory to identify the type of fault.

#### 4.5.2 Prior cases and rules

Prior cases in condition monitoring are analogous to experiences stored in human memory. They serve as a repository of information influencing decision-making process in similar future situations. The choice of case descriptors and their storage format depends on the type of information utilized. Typically, numerical case representations are stored in data structures like arrays, lists, or matrices in computer memory. Each element of the case tuple corresponds to a feature descriptor. Indexing may be employed to effectively search for matches between cases.

In the cyber domain, a rule refers to a set of predefined directive or instruction used within analytics to produce intended results. These rules are often implemented within different modules and submodules of decision-making frameworks. Rules define the boundaries of actions, and serve as a means of regulating behavior, ensuring consistency, and maintaining order and stability in decision-making. In this framework, software libraries, both built-in and user-defined functions, and data analysis algorithms can be regarded as rules.

#### 4.5.3 Domain knowledge

Domain knowledge refers to a comprehensive grasp of a particular field. Every area of study encompasses one or more domains where analysis and exploration are conducted. The research problem in the implementation of this proposed framework is how domain knowledge can be integrated into deliberate decision-making. The domain knowledge consists of three dimensions: knowledge discipline, knowledge representation, and knowledge integration. In any discipline, knowledge is stored in various forms, such as algorithms, mathematical equations, simulation results, logic rules, knowledge graphs, and probabilistic correlations, regardless of the field. Concerning knowledge integration, our aim is to achieve all the advantages associated with condition monitoring, including life extension, reduction of operational costs, and attainment of higher standards/accuracy in production, among others.

### 4.6 Maintenance decision

We face various types of maintenance decisions at different observation points. In situations requiring prompt decision-making, extensive experience plays a key role. In such cases, certain decisions are genuinely automatic, requiring no deliberation. Conversely, when ample time allows for optimal decision-making conditions, advanced analytics, in addition to prior experience, guide the decision-making process. It is crucial to comprehend how the appropriate strategy is chosen to align with business objectives in this scenario.

Like the three modes of information processing in decision-making, we have proposed three types of maintenance decisions: reflexive decision, procedural decision, and deliberate decision, as shown in Figure 2. Reflexive decisions are automatic. Therefore, there is no role for data analytics. These decisions are solely decided by data measurement of sensors into protective action as a reflexive response. Like the two modes of thinking, we have proposed two types of decision-making: procedural decision focused on safety and security, which is quick and relies on a simple approach, and a thoughtful decision centered on business values, which is detailed, comprehensive, and considers various aspects from different perspectives.

There are two primary computational paradigms to formally model decision-making under uncertainty: statistical reasoning and probabilistic reasoning. The procedural analytics relies on statistical reasoning, while the deliberate analytics utilizes probabilistic resoning. It's commonly believed that human decision-making involves a blend of three fundamental techniques: analogies (case-based deduction), rule-based deduction, and probabilistic inference. Within procedural analytics, we categorize models into two sub-techniques: analogies (case-based) (Dhungana, 2024a) and rule-based methods (Dhungana, 2024b).

Procedural decisions are well-defined and structured, meaning all inputs, outputs, and internal procedures are known and specified. In contrast, deliberate decisions are unstructured because they are either new, complex, or rare and lack prior experience. By comparing the decision-making problem from the perspectives of computer science and information technology (Marko, 2009), we classify deliberate decisions as strategic, procedural decisions as tactical, and reflex decisions as operational. By comparing model-based and model-free dichotomy for decision making behavior (Hasz and Redish, 2018), we put model free decision making behavior as deliberate analytics, and model based decision making behavior as procedural analytics.

Within deliberate analytics, Expected Utility Theory (EUT) is widely regarded as the preferred method for making prognostic decisions in uncertain situations. It provides an analytical quantitative framework to determine the most favorable decisions, considering potential outcomes and the probabilities of each structural state. To implement EUT, the utility function is needed, i.e., its values for any consequence that may result from any act. The EUT requires the decision-maker to think in hypothetical or counterfactual terms. In EUT new information is modeled as an event, i.e., a subset of states, which has obtained. The model is restricted to this subset, and the probability is updated according to Bayes' rule. Bayesian model coupled with expected utility maximization standing out as the most prominent approach (Cappello et al., 2016).

**Figure 5:**
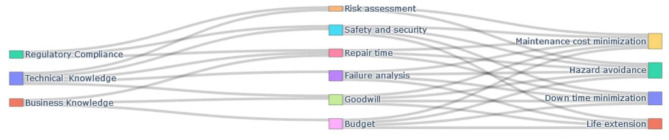
A Sankey diagram of knowledge fusion for the deliberative maintenance decision.

Technical insights alone are insufficient for guiding strategic maintenance decisions in industrial systems. Effective decision-making must also incorporate factors such as regulatory compliance related to safety and risk, as well as adherence to environmental standards. Additionally, business considerations-including cost-benefit analysis, operational efficiency, and organizational goodwill-are essential for aligning maintenance strategies with broader enterprise goals as in Figure 5.

## 5 Empirical studies and discussion

Empirical studies is carried out at the fault identification level of CM using procedural analytics applied to vibration data obtained from bearing degradation tests. The classification methodology integrates both case-based and rule-based analytical techniques to facilitate rapid identification and categorization of bearing faults by interpreting extracted vibration features as diagnostic information. This approach leverages memory-associated classification mechanisms, where case-based classification relies on previously encountered fault patterns, and rule-based classification utilizes predefined logical rules. Both methods contribute to the quick differentiation of bearing health states, encompassing normal operating conditions as well as specific fault types such as inner raceway faults, outer raceway faults, and ball defects.

### 5.1 Datasets

This study employs the 12k Drive End Bearing vibration data from the Case Western Reserve University (CWRU) bearing dataset for fault analysis and detection. Specifically, samples with a fault diameter of 0.021”, a motor load of 3 HP, and a motor speed of 1,730 RPM are used to validate the proposed approach experimentally. The CWRU dataset provides comprehensive ball-bearing test data, including both healthy and faulty conditions, and serves as a widely recognized benchmark in bearing fault diagnosis research (Smith and Randall, 2015).

The experiments were conducted using a 2-horsepower Reliance Electric motor, with vibration data collected via accelerometers mounted at both proximal and distal positions relative to the motor bearings. Faults with a diameter of 0.021 inches and a depth of 0.011 inches were systematically introduced into three specific bearing locations: the inner raceway (IR), the ball, and the outer raceway (OR). These faulty bearings were reinstalled into the motor, and vibration signals were recorded at a consistent motor speed of 1,730 RPM under a 3 HP load, with a sampling rate of 12,000 samples per second. The drive end bearing under investigation is a 6205-2RS JEM SKF deep groove ball bearing, characterized by an inner diameter of 0.9843”, an outer diameter of 2.0472”, a thickness of 0.5906”, a ball diameter of 0.3126”, and a pitch diameter of 1.537”. The dataset includes conditions for normal bearings and those with single-point defects at the drive end. Vibration data were acquired using accelerometers mounted at the 12 o'clock position on the bearing housing with magnetic bases to ensure stable placement. All data are stored in MATLAB (*.mat) format and accompanied by detailed documentation of the experimental setup, which is available through the official CWRU website.^1^

### 5.2 Experimental settings and evaluation metrics

The vibration measurement data was divided into segments of 1,000 data points, corresponding to 0.0833 seconds, covering at least two full bearing rotations. The healthy sample of healthy dataset was labeled into normal class and specific bearing defects such as inner race, outer race, and ball defects categories, with one for healthy samples and the rest for faulty ones, differentiated by fault diameter and location. Each vibration file contained over 120,000 measurements for faulty classes and over 480,000 for healthy ones. To balance the classes, we used 120 samples per class together 480 samples. The dataset was used for a 4-category classification-based fault location on bearing parts.

Features like peak-to-peak, dominant frequency and harmonics of vibration are extracted as equivalent to information extraction from raw vibration data for fault diagnosis. It reads a dataset of vibration signals, calculates key signal features-peak value, dominant frequency, and number of harmonics-for each signal sample. The dominant frequency and harmonics are computed using the Fast Fourier Transform (FFT), focusing on the most prominent frequency components. These extracted features are then compiled along with the corresponding fault labels. All analytics were executed on a personal computer with an Intel(R) Core(TM) i9-10900X CPU @ 3.70 GHz and 32 GB of memory, using Jupyter Notebook.

Standard performance metrics for classification tasks accuracy, precision, recall, and F1 score are employed as in Equations 1–4 to evaluate fault identification based on procedural analytics (Sun et al., 2020). Accuracy measures the overall correctness of the model by calculating the ratio of correctly predicted instances to the total instances. Precision indicates how many of the predicted positive cases are actually correct, reflecting the model's ability to avoid false alarms. Recall measures the model's ability to detect all actual positive cases, showing how well it captures true faults. *F*1 Score is the harmonic mean of precision and recall, providing a balanced evaluation when both false positives and false negatives are important.


(1) $$\begin{array}{l} \text{Accuracy}=\frac{TP+TN}{TP+TN+FP+FN} \end{array}$$

(2) $$\begin{array}{l} \text{Precision}=\frac{TP}{TP+FP} \end{array}$$

(3) $$\begin{array}{l} \text{Recall (Sensitivity)}=\frac{TP}{TP+FN} \end{array}$$

(4) $$\begin{array}{l} \text{F1 Score}=2\times \frac{\text{Precision}\times \text{Recall}}{\text{Precision}+\text{Recall}} \end{array}$$

where: *TP* = True Positives (correctly predicted positive cases), *TN* = True Negatives (correctly predicted negative cases), *FP* = False Positives (negative cases incorrectly predicted as positive), *FN* = False Negatives (positive cases incorrectly predicted as negative).

### 5.3 Results

**Figure 6:**
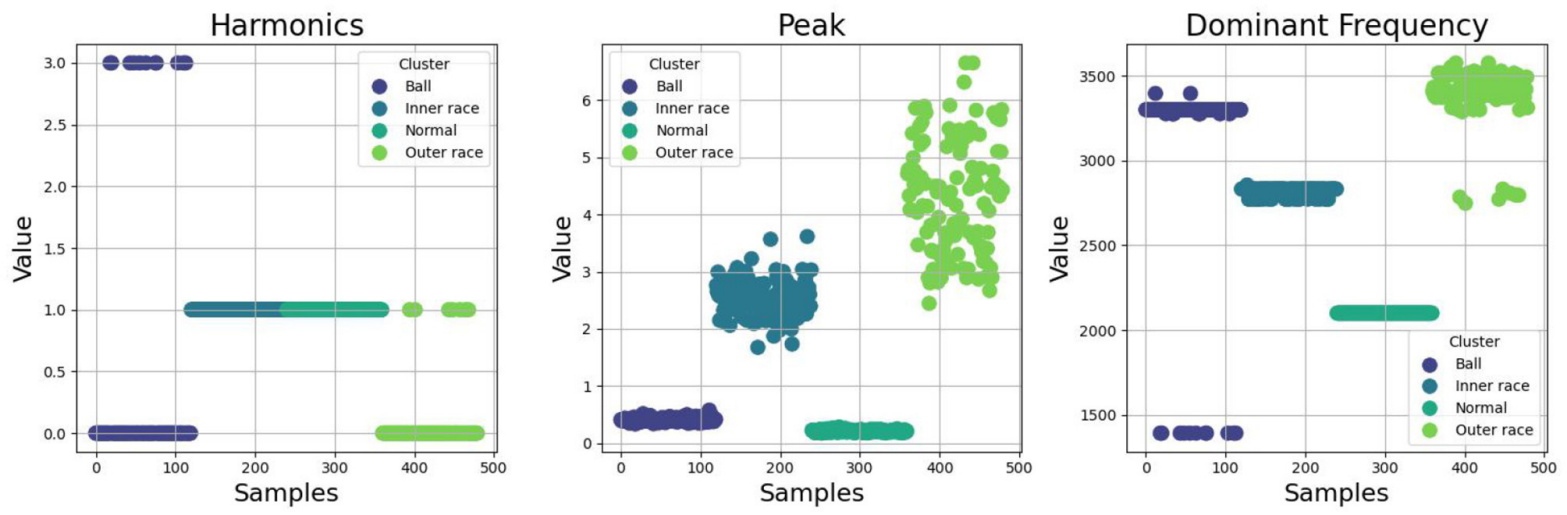
Nature of feature distributions of three different failure parts along with normal bearing.

To construct representative cases and interpretable classification rules, feature distributions of vibration data were visualized for three distinct bearing fault types-inner race, outer race, and ball defects-alongside normal bearing conditions. The selected features include the peak-to-peak amplitude of the vibration signal, the dominant frequency component, and the number of harmonics present in each measurement sample. Figure 6 shows an intuitive understanding of feature behavior across different fault types, enabling the formulation of discriminative and explainable classification rules.

#### 5.3.1 Case-based classification

As in Case-Based Decision Making (CBDM), cases are represented as combinations of problem descriptions, actions, and outcomes, with learning achieved through the storage of real instances in memory. Experimental verification of case-based RMS prediction under varying speeds-both increasing and decreasing-was presented in Dhungana (2024a). Similarly, in this work, we formulate a case using three key features as in Equation 5:


(5) $$\begin{array}{l} \text{Case tuple }C=\left(P,D,H\right) \end{array}$$

where: *C* is the case, *P* is the peak, *D* is the dominant frequency, and *H* is the harmonic.

To ensure that each feature contributes equally to case formation without bias from differing magnitudes, we normalize the feature using MinMaxScaler. The four distinct fault categories are then treated as cluster centroids by computing the mean values of each fault class's three key features: Peak, dominant frequency, and harmonics. These mean values are rounded to four decimal places to maintain precision while simplifying interpretation. This approach allows for a clear comparison of feature distributions across different fault types, supporting further classification. Therefore, the cases of four types of bearing condition is represented as cases as tuple and their respective value of each baseline fault category is represented as

Normal case *C*_1_: (0.036, 0.7936, 0.0917),

Ball fault case *C*_2_: (0.3642, 0.6544, 0.3333),

Inner race fault case *C*_3_: (0.0055, 0.3242, 0.3333),

Outer race fault case *C*_4_: (0.6307, 0.9144, 0.0194).

To retrieve similar cases, a similarity metric Sim(*C*_*new*_, *C*_*i*_) is used to compare the new case *c*_*new*_ with baseline cases *C*_*i*_. We use Euclidean Distance as the similarity measure as shown in Equation 6. The baseline case with the smallest distance to the new case is selected as the matched case for fault classification.


(6) $$\begin{array}{l} \text{Sim}\left(C_{new},C_{i}\right)=\text{Euclidean Distance}\left(d\right)\\ =\sqrt{\overset{n}{\underset{j=1}{\sum }}{\left(C_{j}^{new}-C_{j}^{i}\right)}^{2}},\text{ for }i=1,2,3,4 \end{array}$$

Case-based classification was tested on 480 samples from the allocated dataset. Each sample was assigned a fault type based on the minimum Euclidean distance to known cases. The evaluation metrics for case-based classification performance are detailed in the first row of Table 5.

**Table 5:** Fault identification from two approcach.

**Procedural analytics**	**Processing time (ms)**	**Accuracy**	**Precision**	**Recall**	**F1_score**
Case based	48.7237	0.9646	0.9671	0.9646	0.9645
Rule based	37.4417	0.9854	0.9862	0.9854	0.9854

#### 5.3.2 Rule-based classification

Rule-based systems are widely used in expert systems, automation, and decision support systems. Industry standards like ISO 10816 and expert knowledge guide establishing the following vibration severity rules.^2^ These rules are typically expressed in the form of “if-then” statements, where specific conditions (the “if” part) trigger corresponding actions or conclusions (the “then” part). Table 6 shows the generic decision matrix based on vibration data. Inspired by the ISO guideline in the context of the CWRU dataset, we customize the rule based on the nature of the feature distributions in Figure 6. This approach reveals the clear boundary for interpretable decision criteria and transparency and supports actionable insights, especially in systems requiring explainable decision-making. Only four rules are sufficient to distinguish the fault types and are made as follows “if-then” statements. The evaluation metrics for rule-based classification performance are detailed in the last row of Table 5.

- **Rule 1:** If the peak value is less than 1 and the dominant frequency falls between 2,000 Hz and 2,500 Hz, then it is in normal condition.
- **Rule 2:** If the peak value is less than 1 and no specific frequency condition is met, then it is ball fault.
- **Rule 3:** If the peak value is greater than 1 and the dominant frequency is between 2,500 Hz and 3,000 Hz, then it is inner-race fault.
- **Rule 4:** If none of the above conditions are satisfied, then it is oute-race fault.

**Table 6:** Decision matrix based on ISO 20816-1:2016 general machine vibration guidelines.

**Vibration level (mm/s)**	**Dominant frequency**	**Presence of harmonics**	**High frequency components**	**Condition**
<2.5	Any	None	None	Normal condition
2.5–4.5	RPM	None	None	Imbalance
4.5–6.5	Any	2X, 3X of RPM	None	Misalignment
>6.5	Any	None	High frequency components (>1 kHz)	Bearing fault
>6.5	BPFO	X, 2X, 3X of BPFO		Outer race
>6.5	BPFI	X, 2X, 3X of BPFI		Inner race
>6.5	BSF	X, 2X, 3X of BSF		Ball
>6.5	FTF	X, 2X, 3X ofFTF		Cage

Table 5 compares the performance of case-based and rule-based approaches for fault identification. The rule-based method outperforms the case-based one in all key metrics, achieving higher accuracy (98.54% vs. 96.46%), precision, recall, and F1-score, while also requiring less processing time (37.44 ms vs. 48.72 ms). This indicates that the rule-based approach is both more accurate and computationally efficient for fault diagnosis tasks. Even with data from 1,000 vibration readings, the fault identification time using pixel intensity-based inference from the trained model takes 1.41 ms on the same computing setup (Dhungana et al., 2025a). In comparison, the rule-based identification consumes only 0.078 ms.

**Figure 7:**
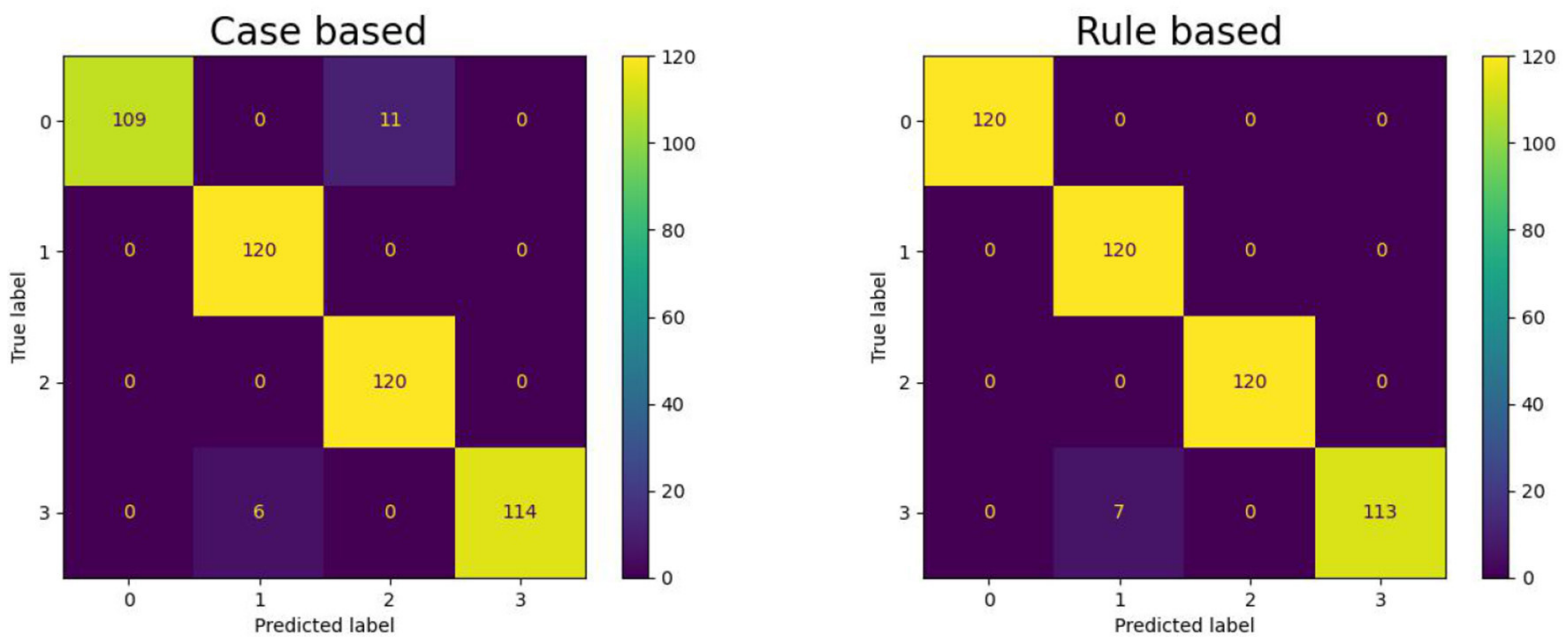
Confusion matrix or fault identification from case base and rule based classification.

The Figure 7 on the left displays the confusion matrix for case-based analytics, while the graph on the right presents the confusion matrix for the results of rule-based analytics. Both methods perform well overall, but the rule-based model shows slightly higher accuracy, especially for Ball faults and Outer race faults, by eliminating misclassifications that occurred in the case-based approach. This suggests that the rule-based method may offer better consistency and generalization for this particular dataset.

### 5.4 Discussion

CBDM offers efficiency by utilizing past experiences to streamline problem-solving, reducing the need to develop solutions from scratch. Its flexibility allows adaptation to novel challenges, while its transparency ensures that decisions remain interpretable and grounded in historical cases. CBDM's effectiveness is contingent on the quality of its case base, the accuracy of similarity measurement, and the complexity of adapting past solutions to new contexts. Formalizing CBDM mathematically enhances its integration into computational systems, such as case-based reasoning (CBR) in AI, improving its scalability and applicability.

An innovative case formation method was introduced, using a single dataset to create three unique feature tuples. While successful in case selection is based on Euclidean distance-based approach requires further refinement for improved accuracy. The case-based classification achieved 96% accuracy using three key features: peak-to-peak value, dominant frequency, and number of harmonics. To further improve accuracy, additional discriminative features-such as spectral kurtosis, RMS, or time–frequency domain descriptors-could be integrated into the case representation. Moreover, optimizing the distance metric or incorporating hybrid approaches combining rule-based logic with case retrieval may enhance classification robustness. We presented empirical studies focused on the retrieval and reuse phases of the case-based classification process without addressing the retention and revision stages. Exploring retention and revision mechanisms will be considered as part of future work to enhance the adaptability of the system.

Rule-based classification offers advantages such as interpretability, simplicity, and real-time decision-making, making it suitable for applications requiring transparent and efficient rule evaluation. Its straightforward structure eliminates the need for complex machine learning models, enabling rapid decision-making in real-time monitoring systems. While rule-based prediction ensures simplicity and interpretability, its limitations include difficulty in capturing complex data relationships and sensitivity to threshold tuning, which may lead to false positives or negatives. Enhancing these systems by integrating machine learning models and employing adaptive thresholds can improve accuracy and adaptability to dynamic operating conditions.

We achieved 99% classification accuracy in a very short time by using only three features to formulate a set of simple yet effective decision rules. In our previous work, a CNN-based approach for real-time fault identification in rolling element bearings achieved a processing time of 6.48 ms per sample (Dhungana et al., 2025a). In comparison, the current rule-based classification method, applied to the same dataset and computational platform, significantly reduces the processing time to just 0.16 ms per sample. These interpretable rules enable fast and accurate fault identification, demonstrating the potential for real-time implementation with minimal computational overhead. Since the dataset does not include fault types such as imbalance or misalignment, our study is limited to a subset of bearing fault conditions. This represents a limitation of the research, as broader fault categories could not be evaluated.

We have demonstrated the experimental verification of our fault identification methodology using vibration data, providing empirical evidence of its applicability in real-world scenarios. This approach not only aids in fault identification but also supports fault detection, quantification, and failure prognostics. However, despite its general applicability across various levels of Condition Monitoring (CM), the methodology faces implementation challenges, such as accuracy variability due to the quality of CM techniques, data consistency, and the complexity of machinery. Scalability issues may arise when applied to large, heterogeneous industrial systems, and sensor placement or data noise could impact the robustness of results. These challenges highlight the need for further refinement, validation in diverse industrial environments, and continuous adaptation to practical limitations.

The proposed methodology is well-suited for industrial systems with extensive sensor networks that generate large volumes of data. It addresses the gap in decision-support tools for engineers by employing biomimetic principles for enhanced data analysis and inference. Given the subjective nature of condition monitoring objectives, which can vary based on system deployment, we do not quantify the strength of connections among these elements. Therefore, the proposed generic procedure empowers researchers and industrial engineers to tailor the weighting according to their specific standards.

## 6 Conclusion

As industrial systems continue to generate increasing volumes of sensor data, the demand for intelligent, interpretable, and resource-efficient condition monitoring solutions grows. In this work, we present a biomimetics-inspired decision-making framework that parallels human cognitive processes, offering a systematic approach to condition management that bridges the gap between raw sensor data and actionable maintenance decisions. Unlike conventional methods, our approach emphasizes the data-information-knowledge hierarchy, focusing on perception, learning, and adaptation rather than material mimicry.

A key contribution of this methodology lies in its ability to support interpretable and adaptive maintenance strategies through continuous rule and case refinement, multi-sensory integration, and energy-efficient processing suitable for constrained environments. Our empirical studies using vibration data validate the framework's practical viability and highlight its flexibility across varying operational scenarios.

The results underscore the importance of simplicity and usability-while the abstract design ensures wide applicability, domain-specific customization remains essential for optimal deployment. Future work will focus on enhancing the computational core through data augmentation, transfer learning, and context-aware analytics, while expanding validation efforts across diverse industrial systems and sensor types. This study provides a novel methodological foundation and a functional roadmap for integrating biomimicry into the next generation of maintenance engineering.

## Data Availability

The data used in this article is publicly available at https://engineering.case.edu/bearingdatacenter/download-data-file.

## Footnotes

^1^*Bearing Data Center | Case School of Engineering*. Available online at: https://engineering.case.edu/bearingdatacenter.

^2^*ISO 20816-1:2016 - Mechanical vibration - Measurement and evaluation of machine vibration - Part 1: General guidelines*. Available online at: https://www.iso.org/standard/63180.html.

## References

[B1] Agnar A. Plaza E. (1994). Case-based reasoning: foundational issues, methodological variations, and system approaches. AI Commun. 7, 39–59. 10.3233/AIC-1994-7104

[B2] Ahmad R. Kamaruddin S. (2012). An overview of time-based and condition-based maintenance in industrial application. Comput. Ind. Eng. 63, 135–149. 10.1016/j.cie.2012.02.002

[B3] Al-Obaidi K. M. Azzam Ismail M. Hussein H. Abdul Rahman A. M. (2017). Biomimetic building skins: an adaptive approach. Renew. Sustain. Energy Rev. 79, 1472–1491. 10.1016/j.rser.2017.05.028

[B4] Atkinson R. C. Shiffrin R. M. (1971). The control of short-term memory. Sci. Am. 225, 82–90. 10.1038/scientificamerican0871-82 5089457

[B5] Avdeenko T. V. Makarova E. S. (2017). Integration of case-based and rule-based reasoning through fuzzy inference in decision support systems. Procedia Comput. Sci. 103, 447–453. 10.1016/j.procs.2017.01.0167869951

[B6] Baskarada S. Koronios A. (2013). Data, information, knowledge, wisdom (DIKW): a semiotic theoretical and empirical exploration of the hierarchy and its quality dimension. Austral. J. Inf. Syst. 18:748. 10.3127/ajis.v18i1.748

[B7] BBC Bitesize (n.d.). How does the nervous system help us respond? - *OCR 21st Century Reflex arc*. Available online at: https://www.bbc.co.uk/bitesize/guides/ztjrng8/revision/3 (accessed August 22, 2024).

[B8] Benyus J. M. (1997). Biomimicry: Innovation Inspired By Nature. London: Harper Collins Publishers.

[B9] Bhushan B. (2009). Biomimetics: Lessons from Nature - an overview. Philos. Trans. R. Soc. A 367, 1445–1486. 10.1098/rsta.2009.0011 19324719

[B10] Blanco E. Zari M. P. Raskin K. Clergeau P. (2021). Urban ecosystem-level biomimicry and regenerative design: linking ecosystem functioning and urban built environments. Sustainability 13:404. 10.3390/su13010404

[B11] Blomkvist A. (2022). Imagination as a skill: a Bayesian proposal. Synthese 200, 1–23. 10.1007/s11229-022-03550-z26941682

[B12] Cappello C. Zonta D. Glisic B. (2016). Expected utility theory for monitoring-based decision-making. Proc. IEEE 104, 1647–1661. 10.1109/JPROC.2015.251154032943027

[B13] Chayaamor-Heil N. Hannachi-Belkadi N. (2017). Towards a platform of investigative tools for biomimicry as a new approach for energy-efficient building design. Buildings 7:19. 10.3390/buildings7010019

[B14] Chemweno P. Pintelon L. Muchiri P. N. Van Horenbeek A. (2018). Risk assessment methodologies in maintenance decision making: A review of dependability modelling approaches. Reliab. Eng. Syst. Safety 173, 64–77. 10.1016/j.ress.2018.01.011

[B15] Côté C. (2019). Reactivity and deliberation in decision-making systems. Game AI Pro 360, 91–102. 10.1201/9780429055058-8

[B16] Cowan N. (1998). Attention and Memory: An Integrated Framework. Oxford: Oxford University Press, 1–336. 10.1093/acprof:oso/9780195119107.001.0001

[B17] Cowan N. (2008). What are the differences between long-term, short-term, and working memory? Prog. Brain Res. 169, 323–338. 10.1016/S0079-6123(07)00020-9 18394484 PMC2657600

[B18] Coyle S. Majidi C. LeDuc P. Hsia K. J. (2018). Bio-inspired soft robotics: Material selection, actuation, and design. Extreme Mech. Lett. 22, 51–59. 10.1016/j.eml.2018.05.00336388255

[B19] Daweri M. S. A. Abdullah S. Ariffin K. A. (2020). A migration-based cuttlefish algorithm with short-term memory for optimization problems. IEEE Access 8, 70270–70292. 10.1109/ACCESS.2020.2986509

[B20] de Jonge B. Scarf P. A. (2020). A review on maintenance optimization. Eur. J. Oper. Res. 285, 805–824. 10.1016/j.ejor.2019.09.047

[B21] de Lange F. P. Heilbron M. Kok P. (2018). How do expectations shape perception? Trends Cogn. Sci. 22, 764–779. 10.1016/j.tics.2018.06.002 30122170

[B22] de Vries K. (2020). You never fake alone. Creative AI in action. Inf. Commun. Soc. 23, 2110–2127. 10.1080/1369118X.2020.1754877

[B23] Del Ser J. Osaba E. Molina D. Yang X. S. Salcedo-Sanz S. Camacho D. . (2019). Bio-inspired computation: where we stand and what's next. Swarm Evolut. Comput. 48, 220–250. 10.1016/j.swevo.2019.04.008

[B24] Dhungana H. (2024a). “Case based decision making in biologically inspired condition management system,” in 7th International Conference on Inventive Computation Technologies, ICICT 2024, 335–339. 10.1109/ICICT60155.2024.1054453827490260

[B25] Dhungana H. (2024b). “Rule-based decision making in biologically inspired condition management system,” in International Conference on Agents and Artificial Intelligence, 1245–1254. 10.5220/001246110000363627534393

[B26] Dhungana H. (2025). A machine learning approach for wind turbine power forecasting for maintenance planning. Energy Inform. 8, 1–25. 10.1186/s42162-024-00459-4

[B27] Dhungana H. Mukhiya S. K. Dhungana P. Karic B. (2025a). “Deep learning-based fault identification in condition monitoring,” in Applications in Electronics Pervading Industry, Environment and Society: APPLEPIES 2024 (Cham: Springer), 418–428. 10.1007/978-3-031-84100-2_50

[B28] Dhungana H. Rykkje T. Lundervold A. S. (2025b). Bearing prognostics using the PRONOSTIA data: a comparative study. IEEE Access 13, 49433–49456. 10.1109/ACCESS.2025.3551772

[B29] Dhungana P. Dhungana H. Khadka S. K. Baskota S. (2025). “Data imputation in iot measurements: challenges and guidelines,” in Applications in Electronics Pervading Industry, Environment and Society: APPLEPIES 2024 (Cham: Springer), 429–438. 10.1007/978-3-031-84100-2_51

[B30] Dhungana P. Singh R. K. Dhungana H. (2024). “Machine learning model for fault detection in safety critical system,” in Applications in Electronics Pervading Industry, Environment and Society. ApplePies 2023. Lecture Notes in Electrical Engineering, ed. F. Bellotti (Cham: Springer), 499–507. 10.1007/978-3-031-48121-5_72

[B31] Dorigo M. Birattari M. Stutzle T. (2006). Ant colony optimization. IEEE Comput. Intell. Magaz. 1, 28–39. 10.1109/MCI.2006.329691

[B32] Emmanouilidis C. Pistofidis P. Bertoncelj L. Katsouros V. Fournaris A. Koulamas C. . (2019). Enabling the human in the loop: Linked data and knowledge in industrial cyber-physical systems. Annu. Rev. Control 47, 249–265. 10.1016/j.arcontrol.2019.03.004

[B33] England S. J. Robert D. (2022). The ecology of electricity and electroreception. Biol. Rev. 97, 383–413. 10.1111/brv.12804 34643022

[B34] Escobar Wolf R. Bouali E. H. Y. Oommen T. (2018). “Risk assessment,” in Encyclopedia of Engineering Geology. Encyclopedia of Earth Sciences Series, eds. P.T. Bobrowsky, and B. Marker (Cham: Springer), 758–761. 10.1007/978-3-319-73568-9_239

[B35] Faris H. Aljarah I. Mirjalili S. Castillo P. A. Merelo J. J. (2016). “EvoloPy: an open-source nature-inspired optimization framework in Python,” in Proceedings of the 8th International Joint Conference on Computational Intelligence, 171–177. 10.5220/0006048201710177

[B36] Filingeri D. (2015). Humidity sensation, cockroaches, worms, and humans: are common sensory mechanisms for hygrosensation shared across species? J. Neurophysiol. 114:763. 10.1152/jn.00730.2014 25318766 PMC4533066

[B37] Formicki K. Korzelecka-Orkisz A. Tański A. (2019). Magnetoreception in fish. J. Fish Biol. 95, 73–91. 10.1111/jfb.13998 31054161

[B38] Fridman L. DIng L. Jenik B. Reimer B. (2019). “Arguing machines: human supervision of black box AI systems that make life-critical decisions,” in Conference on Computer Vision and Pattern Recognition Workshops (CVPRW), 1335–1343. 10.1109/CVPRW.2019.00173

[B39] Fu S. C. Zhong X. L. Zhang Y. Lai T. W. Chan K. C. Lee K. Y. . (2020). Bio-inspired cooling technologies and the applications in buildings. Energy Build. 225:110313. 10.1016/j.enbuild.2020.11031337555249

[B40] Gallego G. Delbruck T. Orchard G. Bartolozzi C. Taba B. Censi A. . (2022). Event-based vision: a survey. IEEE Trans. Pattern Anal. Mach. Intell. 44, 154–180. 10.1109/TPAMI.2020.3008413 32750812

[B41] Gao Z. Shi Q. Fukuda T. Li C. Huang Q. (2019). An overview of biomimetic robots with animal behaviors. Neurocomputing 332, 339–350. 10.1016/j.neucom.2018.12.07140007617

[B42] Gerstenberg T. (2022). What would have happened? Counterfactuals, hypotheticals and causal judgements. Philos. Trans. R. Soc. B 377:1866. 10.1098/rstb.2021.0339 36314143 PMC9629435

[B43] Gilboa I. Schmeidler D. (2001). A Theory of Case-Based Decisions. Cambridge: Cambridge University Press, first edition. 10.1017/CBO9780511493539

[B44] Gracheva E. O. Ingolia N. T. Kelly Y. M. Cordero-Morales J. F. Hollopeter G. Chesler A. T. . (2010). Molecular basis of infrared detection by snakes. Nature 464, 1006–1011. 10.1038/nature08943 20228791 PMC2855400

[B45] Gupta V. Jain R. Meena M. L. Dangayach G. S. (2018). Six-sigma application in tire-manufacturing company: a case study. J. Ind. Eng. In. 14, 511–520. 10.1007/s40092-017-0234-6

[B46] Hasz B. M. Redish A. D. (2018). Deliberation and procedural automation on a two-step task for rats. Front. Integr. Neurosci. 12:30. 10.3389/fnint.2018.00030 30123115 PMC6085996

[B47] Hawkins J. (2021). A Thousand Brains: A New Theory of Intelligence. New York: Basic Books.

[B48] He Q. Wang S. Liu T. Liu C. Liu X. (2024). Enhancing measurement precision for rotor vibration displacement via a progressive video super resolution network. IEEE Trans. Instrum. Meas. 73, 1–13. 10.1109/TIM.2024.3381722

[B49] Hedden B. (2023). Counterfactual decision theory. Mind 132, 730–761. 10.1093/mind/fzac060

[B50] Höfler M. (2005). Causal inference based on counterfactuals. BMC Med. Res. Methodol. 5, 1–12. 10.1186/1471-2288-5-28 16159397 PMC1239917

[B51] Hou J. Jiang H. Wan C. Yi L. Gao S. Ding Y. . (2022). Deep learning and data augmentation based data imputation for structural health monitoring system in multi-sensor damaged state. Measurement 196:111206. 10.1016/j.measurement.2022.111206

[B52] Johnson J. G. Busemeyer J. R. (2014). “Rule-based decision field theory: A dynamic computational model of transitions among decision-making strategies,” in The Routines of Decision Making, 3–20.

[B53] Kahneman D. (2011). Thinking, Fast and Slow. New York: Farrar, Straus and Giroux, 499.

[B54] Kandukuri S. T. Klausen A. Karimi H. R. Robbersmyr K. G. (2016). A review of diagnostics and prognostics of low-speed machinery towards wind turbine farm-level health management. Renew. Sustain. Energy Rev. 53, 697–708. 10.1016/j.rser.2015.08.061

[B55] Katoch S. Chauhan S. S. Kumar V. (2021). A review on genetic algorithm: past, present, and future. Multimed. Tools Appl. 80, 8091–8126. 10.1007/s11042-020-10139-6 33162782 PMC7599983

[B56] Kaveh M. Mesgari M. S. (2023). Application of meta-heuristic algorithms for training neural networks and deep learning architectures: a comprehensive review. Neural Proc. Lett. 55, 4519–4622. 10.1007/s11063-022-11055-6 36339645 PMC9628382

[B57] Kevin C. (2018a). Learning theories: behaviorism. Radiol. Technol. 90, 172–175. 30420574

[B58] Kevin C. (2018b). Learning theories: cognitivism. Radiol. Technol. 90, 176–179. 30420575

[B59] Kevin C. (2018c). Learning theories: constructivism. Radiol. Technol. 90, 180–182. 30420576

[B60] King H. Ocko S. Mahadevan L. (2015). Termite mounds harness diurnal temperature oscillations for ventilation. Proc. Nat. Acad. Sci. 112, 11589–11593. 10.1073/pnas.1423242112 26316023 PMC4577200

[B61] Klapproth F. (2008). Time and decision making in humans. Cogn. Affect. Behav. Neurosci. 8, 509–524. 10.3758/CABN.8.4.509 19033245

[B62] Kumari R. Naick B. K. (2022). “Reliability assessment of distribution system considering protection coordination,” in 2022 2nd International Conference on Emerging Frontiers in Electrical and Electronic Technologies, ICEFEET 2022. 10.1109/ICEFEET51821.2022.9847765

[B63] Lee S. Bain P. A. Musa A. J. Baker C. Li J. (2024). A causal network-based markov decision process model for intervention planning. IEEE Trans. Autom. Sci. Eng. 21, 706–720. 10.1109/TASE.2022.3228643

[B64] Lieder F. Griffiths T. L. (2020). Resource-rational analysis: Understanding human cognition as the optimal use of limited computational resources. Behav. Brain Sci. 43:e1. 10.1017/S0140525X1900061X 30714890

[B65] Lin C. H. Erickson T. W. Fishel J. A. Wettels N. Loeb G. E. (2009). “Signal processing and fabrication of a biomimetic tactile sensor array with thermal, force and microvibration modalities,” in 2009 IEEE International Conference on Robotics and Biomimetics, ROBIO, 129–134. 10.1109/ROBIO.2009.5420611

[B66] Lin G. Liang J. Qian Y. (2015). An information fusion approach by combining multigranulation rough sets and evidence theory. Inf. Sci. 314, 184–199. 10.1016/j.ins.2015.03.051

[B67] Mahadevan S. (2018). “Imagination machines: a new challenge for artificial intelligence,” in Proceedings of the AAAI Conference on Artificial Intelligence, 7988–7993. 10.1609/aaai.v32i1.12214

[B68] Marko B. (2009). Decision making: a computer-science and information-technology viewpoint. Interdisc. Descr. Complex Syst. 7, 22–37.

[B69] Mikulak R. J. McDermott R. Beauregard M. (2017). The Basics of FMEA. New York: Productivity Press.

[B70] Minda J. P. (2020). The Psychology of Thinking: Reasoning, Decision-Making and Problem-Solving. New York: SAGE Publications Ltd - Torrossa.

[B71] Murata T. (1989). Petri nets: properties, analysis and applications. Proc. IEEE 77, 541–580. 10.1109/5.24143

[B72] Newton K. C. Gill A. B. Kajiura S. M. (2019). Electroreception in marine fishes: chondrichthyans. J. Fish Biol. 95, 135–154. 10.1111/jfb.14068 31169300

[B73] Paul L. A. (2014). Transformative Experience. Oxford: Oxford University Press. 10.1093/acprof:oso/9780198717959.001.0001

[B74] Pearl J. (2019). The seven tools of causal inference, with reflections on machine learning. Commun. ACM 62, 54–60. 10.1145/3241036

[B75] Peterson J. L. (1981). Petri Net Theory and the Modeling of Systems. Upper Saddle River: Prentice Hall PTR, 290.

[B76] Qiu C. Li B. Liu H. He S. Hao C. (2022). A novel method for machine tool structure condition monitoring based on knowledge graph. Int. J. Adv. Manuf. Technol. 120, 563–582. 10.1007/s00170-022-08757-5

[B77] Ristoski P. Paulheim H. (2016). Semantic Web in data mining and knowledge discovery: a comprehensive survey. J. Web Semant. 36, 1–22. 10.1016/j.websem.2016.01.001

[B78] Rowley J. (2007). The wisdom hierarchy: representations of the DIKW hierarchy. J. Inf. Sci. 33, 163–180. 10.1177/0165551506070706

[B79] Saremi S. Mirjalili S. Lewis A. (2017). Grasshopper optimisation algorithm: theory and application. Adv. Eng. Softw. 105, 30–47. 10.1016/j.advengsoft.2017.01.004

[B80] Semenoglou A. A. Spiliotis E. Assimakopoulos V. (2023). Data augmentation for univariate time series forecasting with neural networks. Pattern Recognit. 134:109132. 10.1016/j.patcog.2022.109132 36306658

[B81] Shafer G. Logan R. (1987). Implementing Dempster's rule for hierarchical evidence. Artif. Intell. 33, 271–298. 10.1016/0004-3702(87)90040-3

[B82] Shao H. Lin J. Zhang L. Galar D. Kumar U. (2021). A novel approach of multisensory fusion to collaborative fault diagnosis in maintenance. Inf. Fusion 74, 65–76. 10.1016/j.inffus.2021.03.008

[B83] Sharma R. K. Sharma P. (2010). System failure behavior and maintenance decision making using, RCA, FMEA and FM. J. Quality Mainten. Eng. 16, 64–88. 10.1108/13552511011030336

[B84] Singh M. Øvsthus K. Kampen A.-L. Dhungana H. (2024a). Development of a human cognition inspired condition management system for equipment. Int. J. Syst. Assur. Eng. Manag. 1, 1–10. 10.1007/s13198-024-02391-y

[B85] Singh M. Øvsthus K. Kampen A. L. Dhungana H. (2024b). Initial fault identification for procedural decision making using biologically inspired condition management system. Mechan. Mach. Sci. 152, 641–657. 10.1007/978-3-031-49421-5_52

[B86] Smith W. A. Randall R. B. (2015). Rolling element bearing diagnostics using the Case Western Reserve University data: a benchmark study. Mech. Syst. Signal Process. 64, 100–131. 10.1016/j.ymssp.2015.04.021

[B87] Stroble J. K. Stone R. B. Watkins S. E. (2009). An overview of biomimetic sensor technology. Sensor Rev. 29, 112–119. 10.1108/02602280910936219

[B88] Sun K. H. Huh H. Tama B. A. Lee S. Y. Jung J. H. Lee S. (2020). Vision-based fault diagnostics using explainable deep learning with class activation maps. IEEE Access 8, 129169–129179. 10.1109/ACCESS.2020.3009852

[B89] Todorovic D. (2008). Gestalt principles. Scholarpedia 3:5345. 10.4249/scholarpedia.5345

[B90] Tuninetti V. Alzugaray R. González J. Valenzuela M. Jaramillo A. Diez E. (2021). Root cause and vibration analysis to increase veneer manufacturing process efficiency: a case study on an industrial peeling lathe. Eur. J. Wood Wood Prod. 79, 951–966. 10.1007/s00107-021-01705-2

[B91] Van Der Meer M. Kurth-Nelson Z. Redish A. D. (2012). Information processing in decision-making systems. Neuroscientist 18, 342–359. 10.1177/1073858411435128 22492194 PMC4428660

[B92] Van Thieu N. Mirjalili S. (2023). MEALPY: an open-source library for latest meta-heuristic algorithms in Python. J. Syst. Architect. 139:102871. 10.1016/j.sysarc.2023.102871

[B93] Verplanken B. M. Vemund Rudi E. (2014). “The measurement of habit,” in The Routines of Decision Making, 231–247.

[B94] Vonrueden L. Mayer S. Beckh K. Georgiev B. Giesselbach S. Heese R. . (2021). Informed machine learning - a taxonomy and survey of integrating prior knowledge into learning systems. IEEE Trans. Knowl. Data Eng. 35, 614–633. 10.1109/TKDE.2021.3079836

[B95] Watson I. Marir F. (1994). Case-based reasoning: a review. Knowl. Eng. Rev. 9, 327–354. 10.1017/S0269888900007098

[B96] Wegst U. G. Bai H. Saiz E. Tomsia A. P. Ritchie R. O. (2014). Bioinspired structural materials. Nat. Mater. 14, 23–36. 10.1038/nmat4089 25344782

[B97] Wiltschko R. Wiltschko W. (2019). Magnetoreception in birds. J. R. Soc. Interf. 16:20190295. 10.1098/rsif.2019.0295 31480921 PMC6769297

[B98] Zhao X. Jia Y. Li A. Jiang R. Song Y. (2020). Multi-source knowledge fusion: a survey. World Wide Web 23, 2567–2592. 10.1007/s11280-020-00811-037177570

[B99] Zio E. (2018). The future of risk assessment. Reliability Eng. Syst. Safety 177, 176–190. 10.1016/j.ress.2018.04.020

